# Incorporating fine‐scale environmental heterogeneity into broad‐extent models

**DOI:** 10.1111/2041-210X.13177

**Published:** 2019-04-08

**Authors:** Laura J. Graham, Rebecca Spake, Simon Gillings, Kevin Watts, Felix Eigenbrod

**Affiliations:** ^1^ Geography and Environment University of Southampton Southampton UK; ^2^ British Trust for Ornithology Thetford UK; ^3^ Forest Research Centre for Ecosystems, Society & Biosecurity Surrey UK; ^4^ Biological and Environmental Sciences University of Stirling Stirling UK

**Keywords:** biodiversity, environmental heterogeneity, landscape ecology, macroecology, scale

## Abstract

1. A key aim of ecology is to understand the drivers of ecological patterns, so that we can accurately predict the effects of global environmental change. However, in many cases, predictors are measured at a finer resolution than the ecological response. We therefore require data aggregation methods that avoid loss of information on fine‐grain heterogeneity.
2. We present a data aggregation method that, unlike current approaches, reduces the loss of information on fine‐grain spatial structure in environmental heterogeneity for use with coarse‐grain ecological datasets. Our method contains three steps: (a) define analysis scales (predictor grain, response grain, scale‐of‐effect); (b) use a moving window to calculate a measure of variability in environment (predictor grain) at the process‐relevant scale (scale‐of‐effect); and (c) aggregate the moving window calculations to the coarsest resolution (response grain). We show the theoretical basis for our method using simulated landscapes and the practical utility with a case study. Our method is available as the grainchanger r package.
3. The simulations show that information about spatial structure is captured that would have been lost using a direct aggregation approach, and that our method is particularly useful in landscapes with spatial autocorrelation in the environmental predictor variable (e.g. fragmented landscapes) and when the scale‐of‐effect is small relative to the response grain. We use our data aggregation method to find the appropriate scale‐of‐effect of land cover diversity on Eurasian jay *Garrulus glandarius* abundance in the UK. We then model the interactive effect of land cover heterogeneity and temperature on *G. glandarius* abundance. Our method enables us quantify this interaction despite the different scales at which these factors influence *G. glandarius* abundance.
4. Our data aggregation method allows us to integrate variables that act at varying scales into one model with limited loss of information, which has wide applicability for spatial analyses beyond the specific ecological context considered here. Key ecological applications include being able to estimate the interactive effect of drivers that vary at different scales (such as climate and land cover), and to systematically examine the scale dependence of the effects of environmental heterogeneity in combination with the effects of climate change on biodiversity.

## 1 INTRODUCTION

A major goal of ecology is to understand the drivers of ecological processes (Begon, Harper, & Townsend, 2006) and the current dramatic decline of biodiversity (Butchart et al., 2010). There is broad agreement that climate is one of the key factors determining the patterns of species richness (Field et al., 2009), and that climate change is a significant major threat to biodiversity (Thomas et al., 2004). It is also widely acknowledged that land use change is the largest current threat to biodiversity (Pimm & Raven, 2000). Climate and land use affect biodiversity additively and interactively (Jetz, Wilcove, & Dobson, 2007; Travis, 2003), but the nature of these interactions is poorly understood (Newbold, 2018). As such, integration of climate and land use change in broadscale biodiversity analyses is required (Brook, Sodhi, & Bradshaw, 2008; Titeux et al., 2017). Additionally, there is increasing recognition that environmental heterogeneity—the complexity, diversity and structure in the environment—is a near‐universal driver of ecological processes (Stein, Gerstner, & Kreft, 2014). Disregarding environmental heterogeneity adversely affects predictions of climate change effects on biodiversity (Luoto & Heikkinen, 2008). Therefore, broadscale modelling needs to include relative, additive and interactive effects of climate, land use and environmental heterogeneity on ecological processes.

Integrating climate and environmental heterogeneity into ecological modelling is complicated by the fact that the spatial resolution at which they affect ecological processes varies greatly (Newbold, 2018). Advances in remote sensing mean that environmental data are increasingly available at fine spatial and temporal resolutions across broad extents (Bush et al., 2017). However, biodiversity data vary in terms of the spatial resolutions and extent at which they are available (Bellard, Bertelsmeier, Leadley, Thuiller, & Courchamp, 2012). Despite large increases in data mobilisation, biodiversity data availability remains poor in many regions (Amano, Lamming, & Sutherland, 2016). For example, broad‐extent data on even the best studied groups in well‐studied regions (e.g. European bird atlases) are typically only reliable at resolutions of 10 km or coarser.

As a result, predictor variables that exert their effects on biodiversity at relatively fine spatial resolutions must be aggregated to the coarser grain of biodiversity response data. While not problematic for regional climatic variables which vary at broad resolutions, this is an issue for factors with finer characteristic scales such as land use, habitat type or topography (Bailey, Boyd, Hjort, Lavers, & Field, 2017). In addition, the inability of coarse‐grain models to adequately represent environmental heterogeneity is a major factor driving inconsistencies between coarse‐grain and fine‐grain predictions of the effects of climate change on biodiversity (Bellard et al., 2012).

Currently, broad‐extent models tend to measure fine‐grain heterogeneity in coarse‐grain models via coarse aggregated measures (Stein & Kreft, 2015; Stein et al., 2014) such as number or percent cover of land cover classes (Algar, Kharouba, Young, & Kerr, 2009; Zuckerberg, Fink, La Sorte, Hochachka, & Kelling, 2016), mean or range of elevation (Graham, Weinstein, Supp, & Graham, 2017; Kreft et al., 2006) and number of topographic features (Bailey et al., 2017). In these ‘direct’ data aggregation approaches, the summary statistic is calculated at the coarser grain by taking, for example, the mean or standard deviation of the finer grain measurements. However, aggregating this way causes a loss of information about the structure of spatial features (Kitron et al., 2006; Turner, O'Neill, Gardner, & Milne, 1989; Wiens, 1989) and means that within‐grain variation for processes that vary, or exert their effects, over a fine scale is lost (Field et al., 2009). More generally, aggregation of data into larger spatial units can change the observed strength and/or direction of a relationship—this is known as the modifiable areal unit problem (MAUP) (Openshaw, 1984). The underlying cause of the MAUP is the smoothing effect of averaging data that are spatially heterogeneous (Gotway & Young, 2002). Therefore, to incorporate fine‐resolution environmental heterogeneity into broad‐extent models effectively, there is a need for data aggregation methods that preserve information about the spatial structure of heterogeneity.

An additional challenge to understanding the effects of environmental heterogeneity on biodiversity is that the scale at which a species responds to the environment varies between species, and if species–environment relationships are modelled at inappropriate scales, we can draw incorrect inferences from our analyses (de Knegt et al., 2010). However, finding the appropriate scale, known as the scale‐of‐effect, can be challenging (Miguet, Jackson, Jackson, Martin, & Fahrig, 2016). Scales‐of‐effect are typically determined using biological understanding of an organism's ecological neighbourhood (Addicott et al., 1987). However, we do not always have a priori understanding of these scales, and many predictions of the factors affecting scales‐of‐effect remain untested (Miguet et al., 2016). In landscape ecology, regressions between the ecological response—measured within a focal patch or point—and the environmental predictor are typically conducted at multiple scales of the predictor, and the scale‐of‐effect is determined as that with the greatest statistical support (Holland, Bert, & Fahrig, 2004). However, this approach is not suitable when the spatial grain of the response is larger than the plausible range of spatial scales at which biodiversity responds to environmental heterogeneity. For example, in atlas data, when a species abundance is measured at a resolution of 10 km, the scale at which the species responds to environmental heterogeneity may be related to a foraging distance of <10 km (Miguet et al., 2016).

There is a literature on improving data aggregation methods; however, this does not link the scale of aggregation to the relevant scale‐of‐effect for the organism or process of interest. Most studies which either examine the effect of data aggregation (Raj, Hamm, & Kant, 2013; Sun, Congalton, Grybas, & Pan, 2017; Wu, 2004) or propose new methods for data aggregation (Frazier, 2014; Gardner, Lookingbill, Townsend, & Ferrari, 2008) focus only on scaling up categorical representations of the landscape (i.e. land cover classes). Their utility is evaluated by their ability to recover fine‐resolution landscape pattern metrics at coarser resolutions. While standardised data aggregation approaches are ideal for studying landscape pattern and investigating drivers of landscape change, they are not appropriate for ecological analysis because an understanding of the scale of the ecological process is not included.

Here, we present a novel method that, for the first time, explicitly links data aggregation to landscape ecological theory. We show that by calculating fine‐scale variation using a moving window at a scale appropriate to the ecological process under study (*sensu* Wiens, 1989), before aggregating to the coarser scale, we obtain critical additional information on environmental heterogeneity (within‐unit variation) over simply calculating variation at the coarser scale. Our approach has important implications as it enables—for the first time—statistically robust testing of hypotheses about the effects of fine‐grain environmental heterogeneity on ecological processes which have been measured using coarse‐grain, broad‐extent data. Specifically, our approach enables (a) systematic testing of the scale dependence of the effects of environmental heterogeneity within broad‐extent models and (b) testing of the interactive and additive effects of environmental heterogeneity within broad‐extent models at ecologically meaningful scales. Unlike most data aggregation approaches, our method can be used with both categorical (e.g. land cover) and continuous data (e.g. elevation).

We first comprehensively test our data aggregation method using simulations to understand (a) the situations in which our data aggregation method provides additional information over direct aggregation methods and (b) the situations in which we are able to identify the correct scale‐of‐effect using our method. Understanding this provides a theoretical basis for our approach and is vital to enable us to make informed a priori predictions of when and why our data aggregation approach is most likely to lead to meaningful new insights. We then test our approach empirically with an example of when environmental heterogeneity may influence an ecological process: relative abundance of Eurasian jay *Garrulus glandarius* across Great Britain. *G. glandarius* requires a combination of forest types: broadleaf for foraging and coniferous for nesting (Holden & Cleeves, 2006). Therefore, the spatial structure and distribution of these habitats within the bird's neighbourhood are likely to influence their abundance. We predict heterogeneity of forest type calculated using our approach would be a stronger predictor than simply calculating coarse‐grain measures at the landscape scale. Moreover, our approach enables us to empirically identify the scale‐of‐effect of heterogeneity of forest type on *G. glandarius* abundance; and 2) assess the interactive and additive effects of heterogeneity at the most relevant scale in combination with other, coarse‐grain predictor variables. We predict that the best fit scale‐of‐effect will be ~1 km because this sits between the home range size (Pons & Pausas, 2008) and average dispersal distance (Paradis, Baillie, Sutherland, & Gregory, 1998) for *G. glandarius*; two factors hypothesised to influence scale‐of‐effect (Miguet et al., 2016).

## 2 MATERIALS AND METHODS

### 2.1 Aggregating environmental heterogeneity at organism‐relevant scales

**Figure 1:**
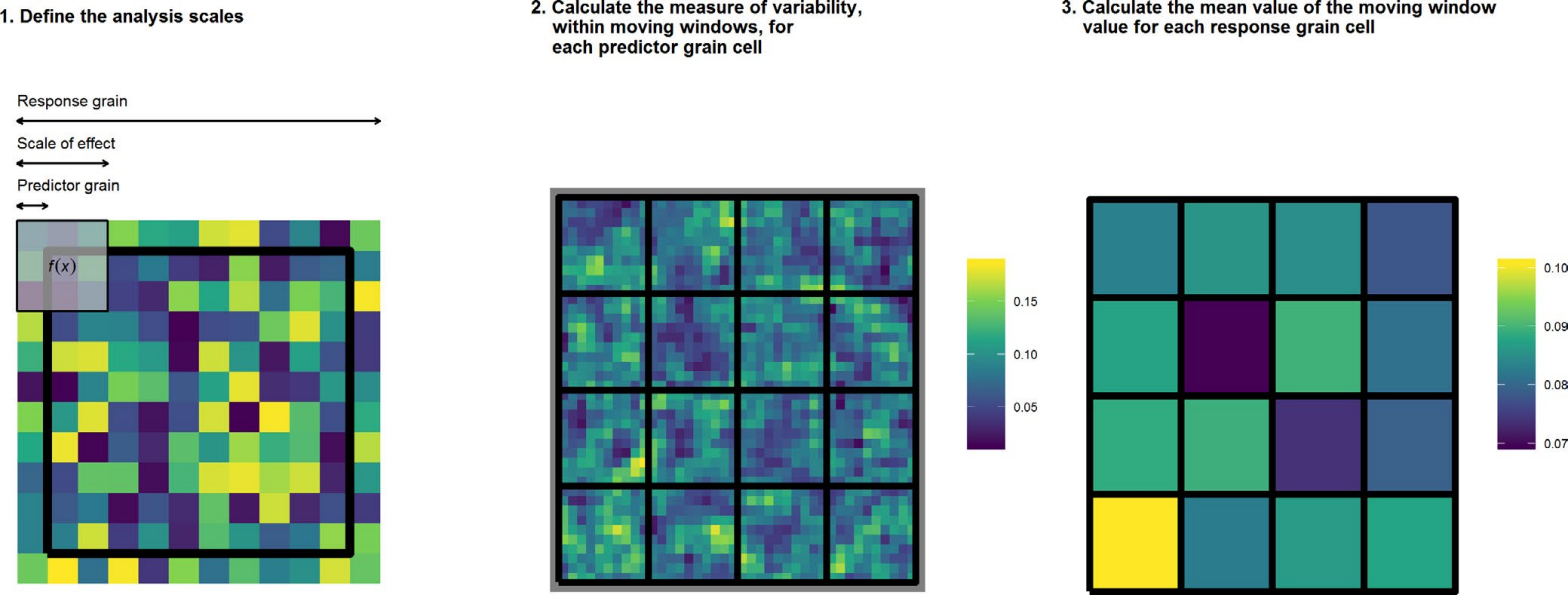
Graphical representation of the moving window data aggregation (MWDA) method. In calculating the MWDA measure, three aspects of scale are considered. Predictor grain is the characteristic spatial scale of the predictor variable, that is, the resolution of the environmental data; scale‐of‐effect determines the appropriate scale of the relationship between predictor and response, for example, an ecological neighbourhood; response grain is the grain of the unit into which you are predicting, that is, the resolution of the response variable. Note that the colour scale is unitless. Yellow cells represent ‘high’ values and dark blue cells ‘low’ values

There are three steps involved in our moving window data aggregation (MWDA) approach: (a) define the appropriate scales for the ecological process; (b) define the appropriate measure of environmental heterogeneity and calculate using a moving window; and (c) summarise the moving window‐based measure at the grain of the response (Figure 1). Our approach is appropriate for any relationship between an environmental factor and an ecological process where the scale‐of‐effect is finer than the scale of analysis. For example, the relationship between landscape structure and occurrence, abundance, fecundity or genetic diversity (Miguet et al., 2016). We have written an r package named grainchanger (Graham, 2019a) to easily implement our method. This package provides the tools to aggregate data from predictor to response resolution through either the MWDA approach (winmove_agg() function) or the direct data aggregation (DDA) approach (nomove_agg() function).

First, we define the scales of analysis: the scale‐of‐effect, response grain and predictor grain (Figure 1, panel 1). The *scale‐of‐effect* is the characteristic spatial scale at which an organism (or ecological process) responds to their environmental context. We can find such scales‐of‐effect by fitting models at multiple scales and selecting the best fitting using information criterion such as Akaike's information criterion (AIC). The scale‐of‐effect of environmental heterogeneity on the ecological process determines the size of the moving window in our method. In our study, we define the size of the scale‐of‐effect as a neighbourhood of *x* units, where *x* represents the distance from the focal cell to the edge of the window and we use the Moore neighbourhood (queen's rule, Moore, 1962).

The *response grain* is the grain at which the ecological process is modelled, and as such the resolution into which the fine‐scale predictor data is being aggregated. This is typically limited by the resolution of the broadscale response data, or is the resolution at which broadscale patterns in the ecological process manifest. For example, for species richness patterns, the grain size should reflect the size of the smallest species range (Rahbek, 2005).

Next, we define a *measure of environmental heterogeneity* that will allow us to quantify environmental heterogeneity within the moving window (set by the scale‐of‐effect of the ecological process of interest). This measure should capture some aspect of the *distribution* of the variable and not its central tendency. For continuous variables, such as elevation or microclimate, simple dispersion measures such as variance, standard deviation and range can be used, as can more complicated measures such as Rao's Q (Rocchini, Marcantonio, & Ricotta, 2017). For categorical variables, such as land cover, number of land cover classes and Simpson's or Shannon's diversity or evenness measures (McGarigal & Marks, 1994) are widely used measures of heterogeneity. The resolution of the analysis within each window should correspond to the thematic resolution at which variability in the environmental variable of interest is captured (*predictor grain*), but may in fact be constrained by the resolution at which the data are measured. We calculate the chosen measure using a moving window with neighbourhood size related to the scale‐of‐effect for each cell of the appropriate grain size in the environmental independent variable. Each predictor grain cell in the raster contains the measure that quantifies the environmental heterogeneity of the surrounding cells within the window (Figure 1, panel 2).

The final step is to aggregate the window‐based measures of environmental heterogeneity from step two to the response grain by calculating a summary statistic (e.g. mean, median, Figure 1, panel 3). This provides a measure that retains more information about spatial characteristics of environmental heterogeneity at the scale‐of‐effect on the ecological process when aggregating to a coarser scale of analysis than direct data aggregation measures.

### 2.2 Aggregating environmental heterogeneity in simulated landscapes

#### 2.2.1 Simulated data

In order to identify the situations under which our MWDA approach is useful, we used simulated datasets to answer two questions: (a) Under what levels of variability in spatial autocorrelation and neighbourhood size (scale‐of‐effect) does the correlation between our MWDA method and DDA break down? (b) In which spatial autocorrelation scenarios can we successfully identify the scale‐of‐effect?

For each dataset, we simulated 1,000 cells at the response grain resolution (10 km × 10 km). For each response grain cell, we simulated landscapes at the predictor grain resolution (25 m × 25 m) using the fractal Brownian motion method (Travis & Dytham, 2004). Using this method, the spatial autocorrelation of a landscape is controlled by the fractal dimension parameter where a value close to zero generates an uncorrelated (i.e. random and highly fragmented) surface, and a value of one a highly autocorrelated (i.e. aggregated and clumped) landscape. Each 25 m × 25 m cell has a continuous value ranging between 0 and 1. We also created a second dataset where each 25 m × 25 m cell has a discrete value between 0 and 4, representing five land cover classes. We created these by generating a vector of class weightings (representing the proportion of each land cover class) and assigning the continuous values to classes based on these weightings. For example, the vector containing 0.5, 0.25 and 0.25 would assign values [0, 0.5] to class 0, values [0.5, 0.75] to class 1, and values [0.75, 1] to class 2. The continuous landscapes represent a fine‐scale continuous environmental variable such as elevation, vegetation indices or microclimate. The categorical landscapes represent fine‐scale categorical environmental variables such as land cover, suitable habitat or soil type. Landscape simulations and classification were done using the NLMR and landscapetools r packages (Sciaini, Fritsch, Scherer, & Simpkins, 2018).

For five scenarios of spatial autocorrelation, we simulated 100 replicate datasets as detailed above. These scenarios were (a) no spatial autocorrelation (fractal dimension = 0.1 for all response grain landscapes); (b) low, varied spatial autocorrelation (fractal dimension in the range 0.1–0.5); (c) varied spatial autocorrelation (fractal dimension in the range 0.1–1); (d) high, varied spatial autocorrelation (fractal dimension in the range 0.5–1); and (e) high spatial autocorrelation (fractal dimension = 1 for all response grain landscapes).

Next, we calculated variability within a moving window at four different neighbourhood sizes: 500 m (1% of response grain), 1 km (4%), 1.5 km (9%) and 3.5 km (49%). To avoid edge effects, we padded each landscape by the neighbourhood size to create the effect of a torus: an infinite surface where cells on one edge neighbour cells on the opposite edge. We calculated variance for the continuous landscapes and Shannon evenness for the categorical landscapes. We calculated Shannon evenness using

(1) $$J^{\prime }=\left(-\sum p_{i}\ln p_{i}\right)/\ln S,$$

*p*_*i*_ is the proportion of land cover class *i* and *S* is the total number of land cover classes (McGarigal & Marks, 1994; Pielou, 1969). In all cases, we aggregated the moving window measure to the response grain by taking the mean across each landscape, resulting in the MWDA measure. Finally, we calculated the same measures using DDA (i.e. by directly calculating the variance and Shannon evenness for each whole landscape).

#### 2.2.2 Correlation between MWDA and DDA approaches

In order to understand the kinds of landscapes where using our data aggregation approach provides different information to standard approaches, we calculated the Spearman correlation between the MWDA and DDA measures for each spatial autocorrelation scenario and neighbourhood size.

#### 2.2.3 Identifying the scale‐of‐effect

In order to understand the specific circumstances (degree of spatial autocorrelation, scale‐of‐effect and signal to noise ratio) under which we can successfully identify the scale‐of‐effect, we also simulated a response variable for each dataset and neighbourhood size. We calculated this response variable as *y*_*w*_ = MWDA_*w*_ + *ɛ* where *y*_*w*_ is the response variable and MWDA_*w*_ is the MWDA measure for neighbourhood size *w* and *ɛ* ∼ *N*(0, σ). We use three levels of σ: low, moderate and high. Low σ represents data with minimal noise and was calculated as the first percentile of the MWDA measure within each spatial autocorrelation scenario and window combination; moderate σ represents data with a moderate amount of noise and was the 10th percentile of the MWDA measure; high σ represents data with a large amount of noise and is the median of the MWDA measure.

For each *y*_*w*_ *,* we fit a univariate linear model with each MWDA_*w*_ as the covariate and use AIC to select the best‐fitting model. We then calculate the % of replicates in which the model containing the correct MWDA_*w*_, and thus scale‐of‐effect, was selected.

### 2.3 Case study: relative abundance of *Garrulus glandarius*

#### 2.3.1 Data

We obtained relative abundance data for Eurasian Jay *G. glandarius* from the British Trust for Ornithology 2007–2011 Bird Atlas (Balmer et al., 2013), which are available at 10 km × 10 km resolution. For this citizen science project, volunteers undertook two 1‐hr timed surveys in at least eight 2 km × 2 km in every 10 km cell in Britain. During these timed surveys, volunteers counted all birds encountered; however, for this study, we convert the counts to presence/absence and determine the proportion of surveyed 2 km × 2 km cells that were occupied for each 10 km cell. These data provide an index of relative abundance for Britain at a resolution of 10 km and have previously been used to map major gradients in abundance (Gibbons, Reid, & Chapman, 1993).

**Figure 2:**
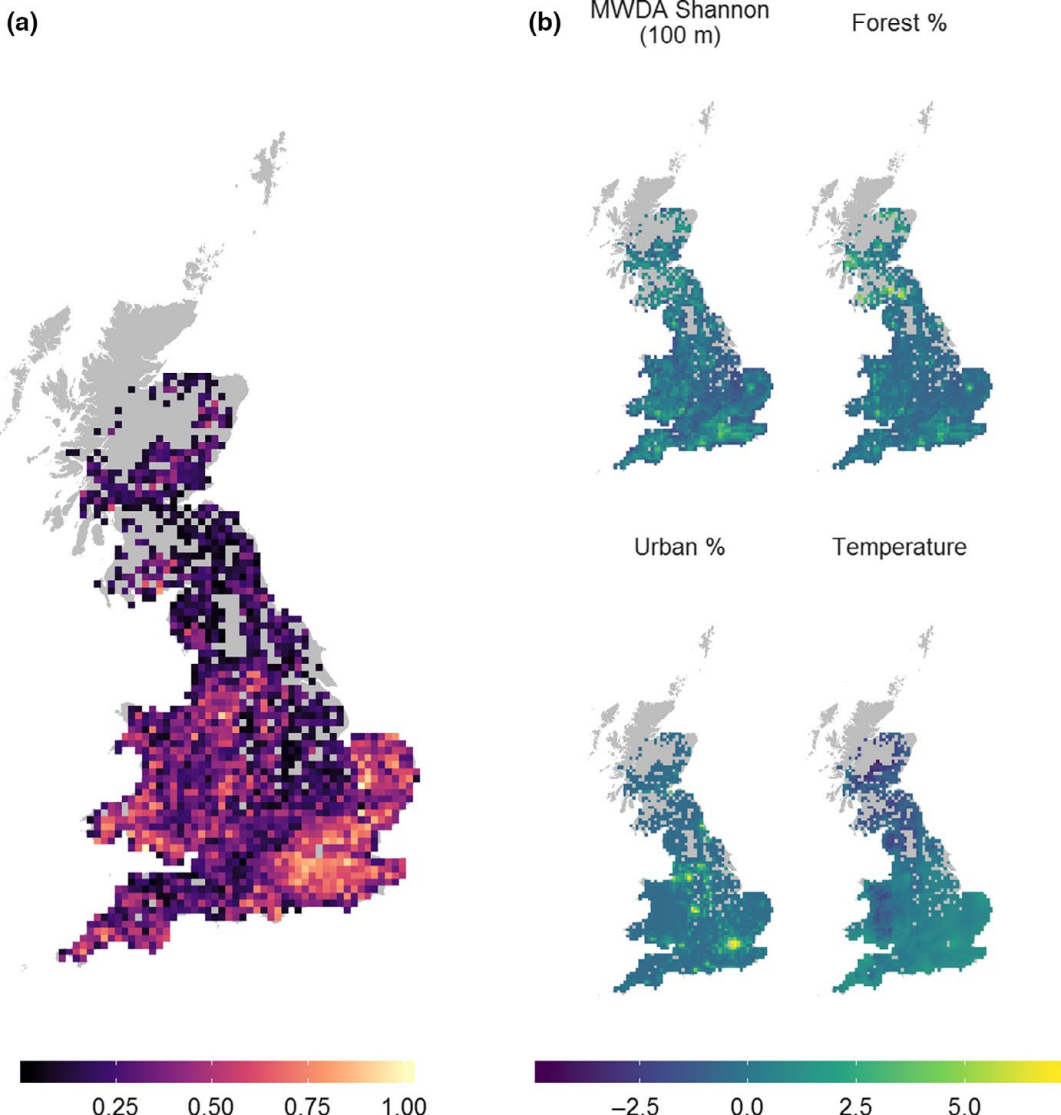
Study site and distribution of *Garrulus glandarius* relative abundance, where a value of one, represented by the lighter colours, is the highest abundance and values approaching zero, represented by the darker colours, are cells with the lowest abundance (a). Mean centred and scaled values of the four predictor variables (b). MWDA Shannon is shown at the 100 m scale‐of‐effect

We obtained land cover data from the 25 m resolution Land Cover Map (LCM) 2007 (Morton et al., 2011), which is the closest match to the 2007–2011 abundance index data. LCM 2007 is a remotely sensed dataset that describes 24 land cover classes. For each 10‐km cell, we calculated forest % and urban % from the LCM 2007 data. We downloaded mean annual temperature (bio1) from WorldClim (Hijmans, Cameron, Parra, Jones, & Jarvis, 2005) at 5 arcminute resolution (~10 km) and matched to the corresponding 10‐km cell. We obtained a full set of response and covariates for *n = *1,719 10‐km cells (Figure 2).

#### 2.3.2 Aggregating environmental heterogeneity

For the *G. glandarius* case study, we aggregated the two forest types in LCM 2007 from 25 m to 10 km resolution using Shannon evenness of broadleaf and coniferous forest as the measure of environmental heterogeneity with both a MWDA and a DDA approach (MWDA Shannon and DDA Shannon respectively). We excluded all other land cover classes from the calculation. In order to identify the appropriate scale‐of‐effect, we calculated MWDA Shannon for six neighbourhood sizes: 50 m, 100 m, 500 m, 1,000 m, 1,500 m and 3,500 m. We aggregated this measure into a response grain of 10 km resolution to match the *G. glandarius* abundance data. For DDA Shannon, we calculated Shannon evenness of forest types for the entire 10 km cell.

#### 2.3.3 Statistical analyses

The *G. glandarius* relative abundance index is a non‐binomial proportion variable. As such, we applied a logit transform to the index and modelled using ordinary linear regression (following Warton & Hui, 2011). We included Shannon, forest %, urban %, temperature and the interaction of temperature with Shannon as covariates. We fit this model for each of the six MWDA measure and the DDA Shannon measure. In order to identify the appropriate scale‐of‐effect, we used AIC and BIC to find the best‐fitting model.

All analyses were performed in r version 3.5.1 (R Core Team, 2018).

## 3 RESULTS

### 3.1 Aggregating environmental heterogeneity in simulated landscapes

#### 3.1.1 Correlation between MWDA and DDA approaches

**Figure 3:**
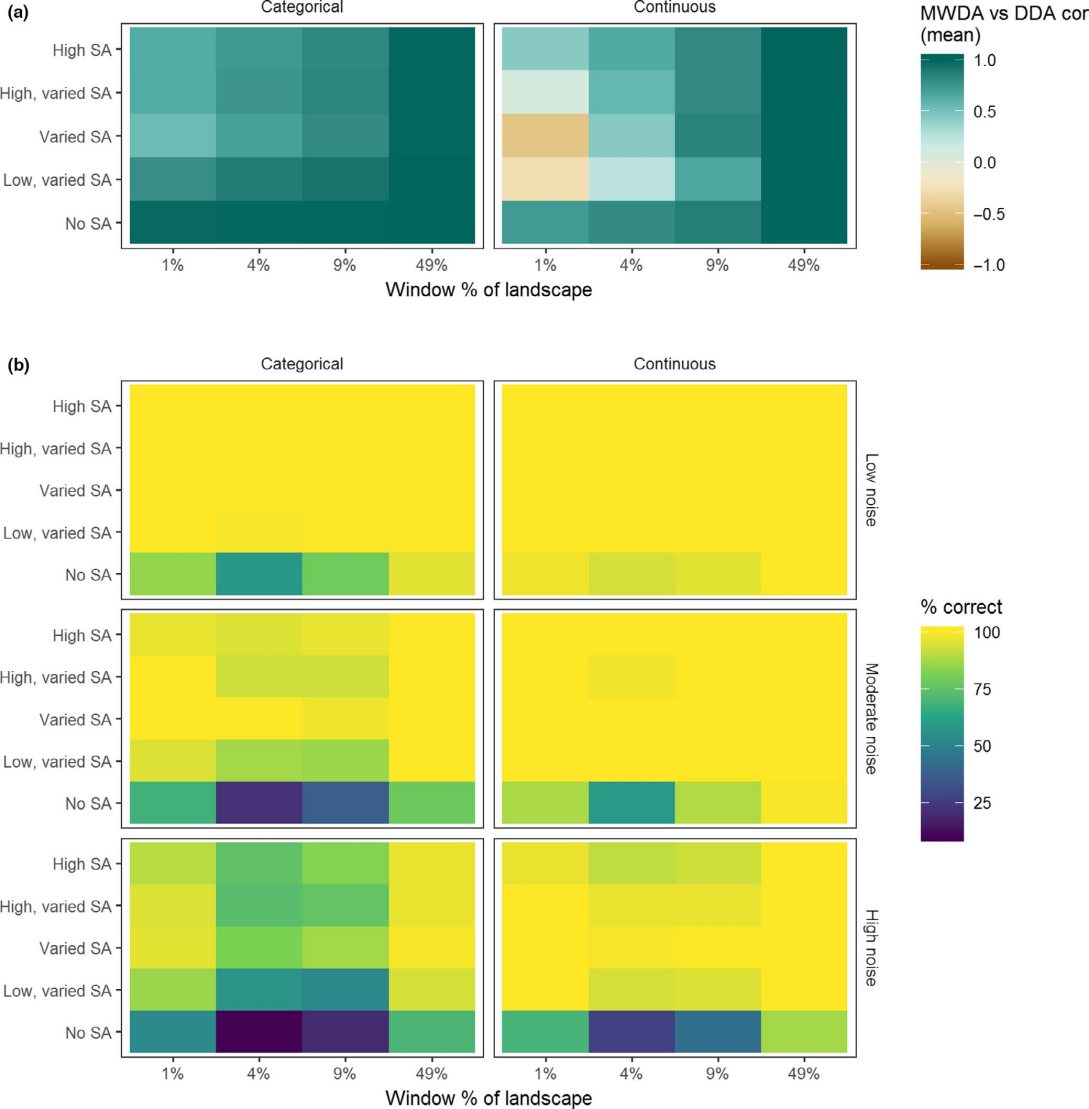
Spearman's ρ between the moving window (MWDA) and direct (DDA) data aggregation measures for each spatial autocorrelation scenario and neighbourhood size (a). Percentage of replicates where the correct scale‐of‐effect was identified for each spatial autocorrelation scenario and neighbourhood size (b)

Correlation between MWDA and DDA measures was lowest for smaller neighbourhood sizes and in varied spatial autocorrelation scenarios (Figure 3a). The pattern was similar between categorical and continuous variables. For the continuous variable, there was a weak negative correlation between MWDA and DDA for the varied, and low, varied spatial autocorrelation scenarios at the smallest neighbourhood size. The reason for this is that high values of spatial autocorrelation result in low MWDA and high DDA, whereas the opposite is the case for low values of spatial autocorrelation (Appendix SI, Figure AI.2).

#### 3.1.2 Identifying the scale‐of‐effect

In most spatial autocorrelation scenarios, neighbourhood sizes and levels of noise in the data, we were able to identify the scale‐of‐effect with reasonable accuracy (Figure 3b). We had the least ability to detect the correct scale‐of‐effect in the no spatial autocorrelation scenario and at intermediate window sizes (4% and 9% of the landscape). We were better able to identify the correct scale‐of‐effect when the predictor variable was continuous, rather than categorical: mean % correct for each noise level ranged from 89.6% in the high noise data to 99.5% in the low noise data for the continuous variable, but 74%–95% for the categorical variable. The scenario in which we had least accuracy in detecting the scale‐of‐effect was the high noise, categorical, no spatial autocorrelation scenario when the window size was 4% of the landscape (15% of replicates correctly identified).

To set the noise levels in the context of an empirical analysis, we calculated the *R* ^2^ values for the model with the correct scale‐of‐effect for each spatial autocorrelation scenario, window size and noise level. The models in the low noise scenario had *R* ^2^ values in the range 0.41–0.91. The models in the moderate noise scenario had *R* ^2^ values in the range 0.1–0.61. The models in the high noise scenario had *R* ^2^ values in the range 0.03–0.28.

### 3.2 Case study: *Garrulus glandarius* abundance

The best‐fitting model judged by both AIC and BIC was that containing the MWDA measure of Shannon diversity with a neighbourhood size of 100 m (Table 1). There was little to distinguish between the MWDA measure at 50 m, 100 m and 500 m (ΔAIC & ΔBIC < 3), suggesting that the true scale‐of‐effect is in this range. The model containing the direct approach to data aggregation (DDA Shannon) had the least support.

**Table 1:** Results of the model comparison. We calculated Shannon diversity of forest cover type using our moving window data aggregation method (MWDA Shannon) and direct data aggregation methods (DDA Shannon). We calculated MWDA Shannon at six scales (defined by the size of the moving window). We then fit seven models of *Garrulus glandarius* abundance changing only the Shannon diversity measure in each. Model performance was evaluated using Akaike's information Criterion (AIC) and Bayesian Information Criterion (BIC). This allowed us to identify the scale at which *G. glandarius* most strongly responds to Shannon diversity of forest cover type

Shannon measurement	AIC	BIC
MWDA Shannon (100 m)	3,975.0	4,013.2
MWDA Shannon (50 m)	3,976.4	4,014.5
MWDA Shannon (500 m)	3,977.4	4,015.6
MWDA Shannon (1,000 m)	3,984.7	4,022.8
MWDA Shannon (1,500 m)	3,991.0	4,029.1
MWDA Shannon (3,500 m)	4,001.9	4,040.1
DDA Shannon	4,005.0	4,043.2

**Figure 4:**
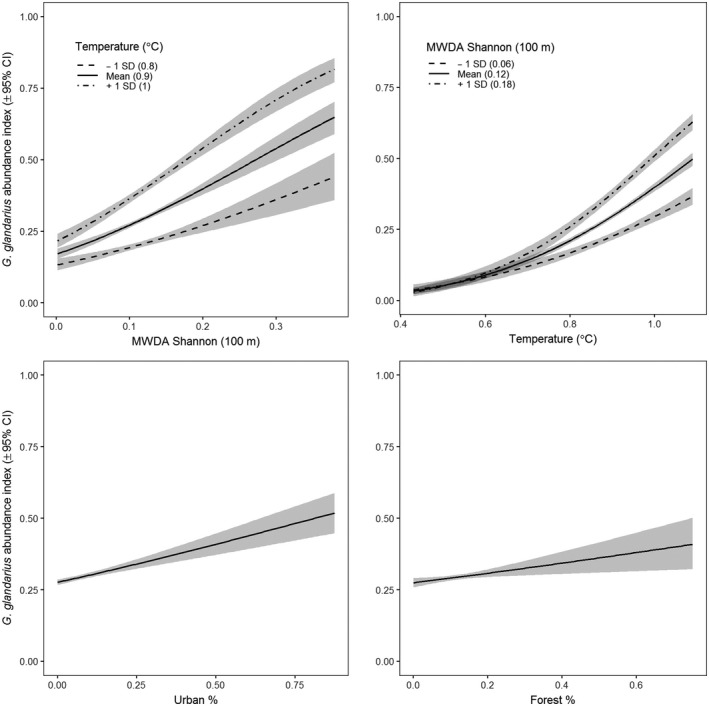
Partial effect plots for *Garrulus glandarius* relative abundance. Interaction plots are shown for MW Shannon and temperature; estimates are provided for ±1 SD of the moderating variable

The model containing MWDA Shannon (100 m) explained a reasonable amount of variation in relative abundance of *G. glandarius* (*R* ^2^ = 0.37). All β coefficients were statistically significant and positive, with the strongest relationships being with MWDA Shannon (100 m) and temperature. We also found a small positive interaction between MWDA Shannon (100 m) and temperature (Figure 4). This interaction was significant for all values of MW Shannon and for all but the lowest temperature values (<6.5°C; calculated using the Johnson–Neyman interval; Johnson & Fay, 1950).

## 4 DISCUSSION

Our results provide a compelling argument for using our novel, three‐step approach to evaluate the effects of fine‐scale environmental heterogeneity on coarse‐scale ecological processes. One key reason why fine‐grain environmental heterogeneity is often considered unimportant within coarse‐grain models is because the within‐unit variability is lost when aggregating to coarse grains (Field et al., 2009). By (a) defining the relevant scale(s) at which heterogeneity matters; (b) using a moving window to calculate environmental heterogeneity; and (c) aggregating to the scale of the response, our approach provides important additional information over existing approaches by capturing such within‐unit variability. Our approach is most useful when an understanding of the effect of heterogeneity on broadscale patterns is the goal of a study, and there is a scale mismatch between the predictor and response data. Additionally, we note that through modelling at the appropriate scale, we gain a greater understanding of mechanism (Levin, 1992; Wiens, 1989), which in turn can increase model transferability and predictive power (Scheiner et al., 2000; Yates et al., 2018).

Our simulations provide the theoretical basis of our data aggregation method. They show that our method is particularly useful in landscapes where within‐unit heterogeneity is variable—or at least where all coarse‐grain cells display high within‐unit heterogeneity. Additionally, our method has greater utility when the scale‐of‐effect is small relative to the response grain. Given most environmental variables display some level of clumping or aggregation (Diniz‐Filho, Bini, & Hawkins, 2003), and this is variable across broadscales (Wu, 2004), it is likely that our method is widely applicable. The low correlation between the MWDA and DDA measures in most scenarios for smaller neighbourhood sizes shows that our method retains more information about spatial structure than direct approaches. This is key information if we are to understand mechanism in ecology (Wiens, 1989) as it means that the MWDA measures capture environmental heterogeneity at the scale at which the ecological process responds to it. Additionally, we showed that measures calculated using our approach are most correlated with and least able to detect the correct scale‐of‐effect when no cells display spatial autocorrelation. It is unlikely that this would occur in reality given most landscapes display some level of within‐unit heterogeneity at coarse resolutions. The smaller correlation between MWDA and DDA measures, and the greater ability to detect the correct scale‐of‐effect at smaller neighbourhood sizes, indicates that it is more important to examine environmental heterogeneity at an appropriate scale in landscapes with a higher level of environmental heterogeneity. This is in agreement with the assertion that changing scale in spatially heterogeneous landscapes can drastically alter conclusions (Scheiner et al., 2000; Wu, 2004) and that the nature of the effect of changing scale depends on the form of heterogeneity in the landscape (Wiens, 1989).

In order to make accurate inferences (Scheiner et al., 2000) and thus gain a greater mechanistic understanding of the effect of environmental drivers (Wiens, 1989), it is key that we understand the appropriate scale at which to model processes. While classical multi‐scale landscape ecology analyses may be employed when the response is measured at point locations (Holland et al., 2004), no analogous methods currently exist when we only have coarse‐grain (i.e. larger than plausible scales‐of‐effect) response data. Using simulations, we showed that our data aggregation approach addresses this, as it is suitable for detecting the correct scale‐of‐effect in most cases for coarse‐grain response data. Our case study demonstrated this in practice, finding that the scale at which forest cover diversity affects *G. glandarius* abundance is in the range of a 50–500 m neighbourhood size. This fits with our prediction that the scale‐of‐effect would be related to the territory size and dispersal distance (Andrén, 1990; Paradis et al., 1998).

A key benefit of being able to include information about fine‐scale environmental heterogeneity in a coarse‐scale model is that we can evaluate the interactive effects of climate and land cover, which is considered a difficult problem and open research area (Bellard et al., 2012; Jetz et al., 2007; Newbold, 2018; Travis, 2003). We found an interactive effect between spatial heterogeneity of forest type and temperature, which suggests that at higher temperatures, the influence of forest diversity on *G. glandarius* abundance is stronger. This means that management for forest diversity will become more important under global climate change, reflecting theoretical and expert‐based expectation (Heller & Zavaleta, 2009).

In addition to deeper understanding of environmental change, our approach also allows us to make conservation relevant conclusions about the scale at which to manage landscapes. The positive effect of forest diversity on relative abundance of *G. glandarius,* when calculated using a moving window with radius 100 m, suggests that management efforts should aim to maintain an even balance of both broadleaved and coniferous forests at this scale in order to benefit populations of *G. glandarius*. The model selection approach allowed us to establish that it was the local‐scale forest diversity driving *G. glandarius* abundance. However, had we calculated forest diversity at the response grain size (10 km x 10 km), we may have concluded that this was an appropriate scale to manage woodlands for *G. glandarius*. Such management may not capture habitat diversity at the relevant scale and lead to inappropriate management (Turner, 1989; Wiens, 1989).

Although using a moving window prior to data aggregation is not necessarily new, studies tend to favour a one‐size‐fits‐all approach by using a 3 × 3‐cell window. For example, topographic measures such as topographic position index and terrain ruggedness index have been calculated in such a way to create multipurpose datasets for use in biodiversity modelling (Amatulli et al., 2018) or to examine the effect of topographic heterogeneity on tropical forest structure and composition (Jucker et al., 2018). Similarly, a global standardized dataset of habitat heterogeneity was calculated using information on adjacent pixels, but without consideration of the scale of the ecological process (Tuanmu & Jetz, 2014). Adopting a one‐size‐fits‐all approach means that the appropriate ecological scale—a key factor in gaining a mechanistic understanding (Levin, 1992; Wiens, 1989)—is not incorporated. Our MWDA method builds on these approaches by explicitly defining the scale at which heterogeneity affects the ecological process within the analysis. Multi‐scale moving window approaches have been used in landscape‐scale analyses using response data available at a point or patch scale (e.g. Bellamy, Scott, & Altringham, 2013; Osborne, Alonso, & Bryant, 2001; Wilson, O'Connell, Brown, Guinan, & Grehan, 2007). Our MWDA approach moves the logic from these two separate literatures forward and provides a method for integrating data that vary at different scales in a broad‐extent analysis. At present, running our MWDA method at very fine grains across global extents is difficult without access to high‐performance computing facilities. However, ongoing improvements in both the efficiency of r for analysing large datasets—which we will implement in future versions of grainchanger—and improvements in computer technology and accessibility mean that this is unlikely to be an issue in the near future.

To fully understand the effect on ecological processes of global change drivers at multiple scales, we must develop an understanding of their interactions and develop modelling approaches which incorporate these interactions at an appropriate scale (Newbold, 2018; Travis, 2003). We have outlined a method for aggregating data on fine‐scale processes that retain information about the underlying spatial structure in environmental heterogeneity at the appropriate scale for the ecological process being analysed. This is crucial if we are to combine spatial data at multiple scales and utilise the growing availability of fine‐resolution environmental data and broad‐extent biodiversity data. For simplicity, we used a generalised linear modelling framework in our analyses. However, variables generated using our data aggregation method could be used as an input to more complex machine‐learning approaches to species distribution (Elith et al., 2006); or to community modelling approaches, such as generalised dissimilarity modelling (Ferrier, Manion, Elith, & Richardson, 2007), and hierarchical modelling of species communities (Ovaskainen et al., 2017). We used model selection to find the scale‐of‐effect; however, this could be found using machine‐learning methods that can handle correlated variables (Bradter, Kunin, Altringham, Thom, & Benton, 2013) or Bayesian approaches (Stuber, Gruber, & Fontaine, 2017) that allow variables generated at multiple scales of effect to be incorporated into one model. Additionally, our method has broader applicability beyond spatial ecology. We focussed here on spatial scale; however, our method could be applied to solve similar issues around temporal scales. Combining data at incompatible spatial and temporal scales is a challenge within many fields including geography, sociology, earth and environmental sciences, agriculture and geology (Gotway & Young, 2002). Our method has the potential to be applied to similar problems in a wider range of contexts and disciplines than those examined here.

## AUTHORS’ CONTRIBUTIONS

L.J.G., F.E. and R.S. conceived the ideas. L.J.G. designed and carried out the package development and statistical analyses. L.J.G. and F.E. wrote the manuscript. All authors discussed the results and contributed to the manuscript.

## Data Availability

All codes to replicate the analyses are available in the supplementary materials and deposited on Zenodo: https://doi.org/10.5281/zenodo.2588304 (Graham, 2019bb *G. glandarius* data are also contained within this Zenodo repository.
