# Supplementary material for: Incorporating fine‐scale environmental heterogeneity into broad‐extent models
Source: Methods Ecol Evol. 2019 Apr 8;10(6):767–78. doi: 10.1111/2041-210X.13177 (PMC6582547; doi:10.1111/2041-210X.13177)
Supplement: Supplementary file 1 [file MEE3-10-767-s001.pdf]

Appendix I: simulated landscapes

Required packages

In the first step, we load all of the required packages. Note that grainchanger (the package developed for this paper) can be installed using:

```
devtools::install_github("laurajanegraham/grainchanger")
```

```
# load required libraries
library(knitr)
library(raster)
library(grainchanger)
library(broom)
library(landscapetools)
library(NLMR)
library(tidyverse)
library(patchwork)
library(cowplot)

# set up plotting options
theme_set(theme_bw(base_size = 8) +
            theme(strip.background = element_blank(),
                  panel.grid.major = element_blank(),
                  panel.grid.minor = element_blank()))
```

Simulated landscapes

1. Simulate study landscapes each with 100 coarse grid cells (each of 65 x 65 fine grid cells) with varying combinations of spatial autocorrelation using `nlm_fbm` from the `NLMR` package. This is the fractal Brownian motion method where `fract_dim` controls the level of spatial autocorrelation in the landscape. A value close to zero is a rough landscape (random), and a value of one is a smooth landscape (spatially autocorrelated). We used 5 combinations of spatial autocorrelation:
   - No spatial autocorrelation: `fractal_dim` = 0.1 for all landscapes
   - Low, varied spatial autocorrelation: `fractal_dim` in range 0.1–0.5
   - Varied spatial autocorrelation: `fractal_dim` in range 0.1–1
   - High, varied spatial autocorrelation: `fractal_dim` in range 0.5–1
   - High spatial autocorrelation: `fractal_dim` = 1 for all landscapes
2. Create categorical landscapes with 5 land cover classes by using `util_classify` with random weights for each class.
3. Use `winmove` from the `grainchanger` package to calculate the moving window measures (MWDA) for each coarse grid cell. For continuous landscapes, this is variance; for categorical landscapes, this is Shannon evenness. We do this for 4 neighbourhood sizes: 500, 1000, 1500, 3500
   - 500 m (1 % of coarse grid cell)
   - 1 km (4 % of coarse grid cell)
   - 1.5 km (9% of coarse grid cell)
   - 3.5 km (49% of coarse grid cell)
4. Calculate the corresponding measures for each coarse grid cell using standard (non-moving window) methods (DDA).

We created 100 replicate study landscapes using the Iridis HPC at University of Southampton. The below code is for one replicate:

```r
library(raster)
library(NLMR)
library(landscapetools)
library(grainchanger)
library(tidyverse)
library(furrr)

plan(multiprocess)

# 1. Create the landscapes and calculate LS and MW measures
# Set up spatial autocorrelation parameters

strt <- Sys.time()
res <- bind_rows(
  tibble(sa_scenario = "No SA", sa_values = rep(0.1, 100)),
  tibble(sa_scenario = "Low, varied SA", sa_values = seq(0.1, 0.5, length.out = 100)),
  tibble(sa_scenario = "Varied SA", sa_values = seq(0.1, 1, length.out = 100)),
  tibble(sa_scenario = "High, varied SA", sa_values = seq(0.5, 1, length.out = 100)),
  tibble(sa_scenario = "High SA", sa_values = rep(1, 100))
) %>%
  # create the continuous and categorical landscapes
  mutate(
    cont_ls = future_map(sa_values, function(x) {
      nlm_fbm(ncol = 400,
              nrow = 400,
              resolution = 25,
              fract_dim = x)
    }),
    # the categorical map has a random proportion between landscapes for each landscape
    cat_wt = future_map(sa_values, function(x) {
      diff(c(0, sort(runif(4)), 1))
    }),
    cat_ls = future_map2(cont_ls, cat_wt, function(x, wt) {
      util_classify(x, weighting = wt)
    }),
    # calculate DDA
    cont_lsm = future_map_dbl(cont_ls, function(x) {
      x %>% raster::values() %>% var
    }),
    cat_lsm = future_map_dbl(cat_ls, function(x) {
      diversity(x, lc_class = 0:4)
    })) %>%
  crossing(window = c(500, 1000, 1500, 3500)) %>%
  mutate(cont_ls_pad = future_map2(cont_ls, window, create_torus),
         cat_ls_pad = future_map2(cat_ls, window, create_torus),
         # calculate MWDA
         cont_mwm = future_map2_dbl(cont_ls_pad, window, function(ls, w) {
           winmove(ls,
                   d = w,
                   type = "rectangle",
                   fun = "var") %>%
```

```
            trim %>%
            raster::values() %>%
            mean
        }),
        cat_mwm = future_map2_dbl(cat_ls_pad, window, function(ls, w) {
          winmove(ls,
                  d = w,
                  type = "rectangle",
                  fun = "shei",
                  lc_class = 0:4) %>%
            trim %>%
            raster::values() %>%
            mean
        })) %>%
  select(-cont_ls, -cat_ls, -cont_ls_pad, -cat_ls_pad)

runtime = difftime(Sys.time(),  strt)
out = list(res = res, runtime = runtime)
save(out, file = tempfile(tmpdir = ".", fileext = ".Rda"))
```

We load all the simulated data:

```
load("results/sims_all_replicates.Rda")

win_label <- round(((2*df$window + 1)^2/10000^2)*100, 1) %>%
  unique() %>%
  paste0("%")

df <- df %>%
  mutate(sa_scenario = factor(sa_scenario,
                              levels = c("No SA",
                                         "Low, varied SA",
                                         "Varied SA",
                                         "High, varied SA",
                                         "High SA")),
         window = factor(window,
                         labels = win_label),
         rep = ceiling((rep %>%
                          as.factor %>%
                          as.numeric)/10)) %>%
  group_by(rep, sa_scenario, window) %>%
  mutate(ls_id = 1:n()) %>%
  ungroup()
```

We use the simulated landscapes to explore two questions:

1. What is the correlation between the moving window and standard aggregations at different levels of spatial autocorrelation and moving window sizes?
2. Can we successfully identify the scale of effect?

Question 1: Correlation between MW and LS

Here we calculate the Spearman's rank correlation between MWDA and DDA for each replicate at each level of spatial autocorrelation and moving window size. The plot below shows the mean and standard deviation

of these correlations (across 100 replicates).

```
corrs <- df %>%
  group_by(rep, sa_scenario, window) %>%
  nest() %>%
  mutate(
    cont_cor = map_dbl(data, function(x) {
    cor.test(x$cont_lsm, x$cont_mwm, method = "spearman")$estimate
  }),
  cat_cor = map_dbl(data, function(x) {
    cor.test(x$cat_lsm, x$cat_mwm, method = "spearman")$estimate}
  )) %>%
  select(-data) %>%
  group_by(sa_scenario, window) %>%
  summarise_at(.vars = vars(cont_cor, cat_cor), .funs = c("mean", "sd")) %>%
  gather(measure, value, -sa_scenario, -window) %>%
  mutate(measure = factor(measure,
                          labels = c("Categorical_mean", "Categorical_sd",
                                     "Continuous_mean", "Continuous_sd"))) %>%
  separate(measure, into = c("ls_type", "measure_type"), sep = "_")

# 1. correlations between MW and LS for diff spatial autocorrelation and window sizes
p1 <- ggplot(corrs %>% filter(measure_type == "mean"),
       aes(x = window, y = sa_scenario, fill = value)) +
  geom_tile() +
  scale_fill_distiller("MWDA vs DDA cor\n(mean)",
                       palette = "BrBG",
                       limits = c(-1, 1),
                       direction = 1) +
  labs(x = "Window % of landscape", y = "") +
  facet_wrap(~ls_type)

p2 <- ggplot(corrs %>% filter(measure_type == "sd"),
             aes(x = window, y = sa_scenario, fill = value)) +
  geom_tile() +
  scale_fill_distiller("MWDA vs DDA cor\n(SD)",
                       palette = "BrBG") +
  labs(x = "Window % of landscape", y = "") +
  facet_wrap(~ls_type)


p1 / p2
```

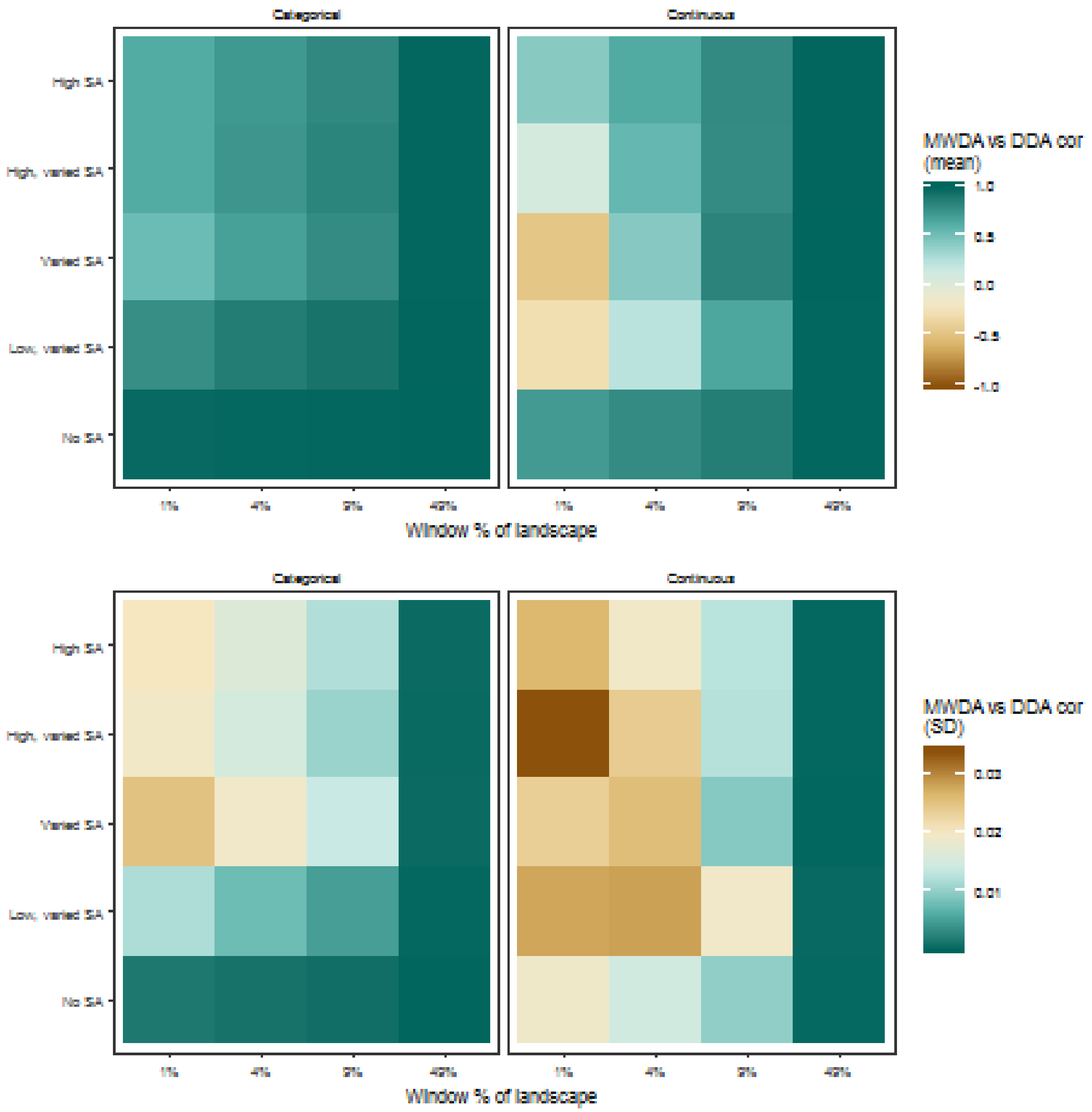

Figure AI.1 Mean correlations between measures calcualted using the moving window (MWDA) and direct (DDA) approaches to data aggregation for continuous and categorical landscapes under 5 different landscape types and 4 different scales-of-effect (top row). Standard deviations of these correlations (bottom row).

- Higher correlation between MWDA and DDA in continuous landscapes
- We even get negative correlation in categorical landscapes for small moving window and variation in spatial autocorrelation.
- MW and LS are highly correlated for the two largest window sizes.
- There is more variability between the amount of correlation in replicates for continuous than categorical landscapes.

Why do we get negative correlations in some cases? The below plots show each spatial autocorrelation scenario and window size combination which results in a negative correlation between MWDA and DDA measures. In the first plot, points are coloured by the spatial autocorrelation value used to generate the landscape. In the second, they are coloured by the replicate ID.

```r
neg_corr <- corrs %>% filter(value < 0) %>%
  inner_join(df) %>%
  filter(rep %in% 1:10)

neg_p1 <- ggplot(neg_corr,
                 aes(x = cont_lsm,
                     y = cont_mwm,
                     colour = sa_values,
                     group = sa_values)) +
  geom_point() +
  scale_fill_continuous(name = "Spatial Autocorrelation") +
  facet_wrap(~ sa_scenario + window) +
  labs(x = "DDA", y = "MWDA")

neg_p2 <- ggplot(neg_corr,
                 aes(x = cont_lsm,
                     y = cont_mwm,
                     colour = rep,
                     group = rep)) +
  geom_point() +
  scale_fill_continuous(name = "Replicate") +
  facet_wrap(~ sa_scenario + window) +
  labs(x = "DDA", y = "MWDA")

neg_p1 / neg_p2 + plot_annotation(tag_levels = "a", tag_suffix = ")")
```

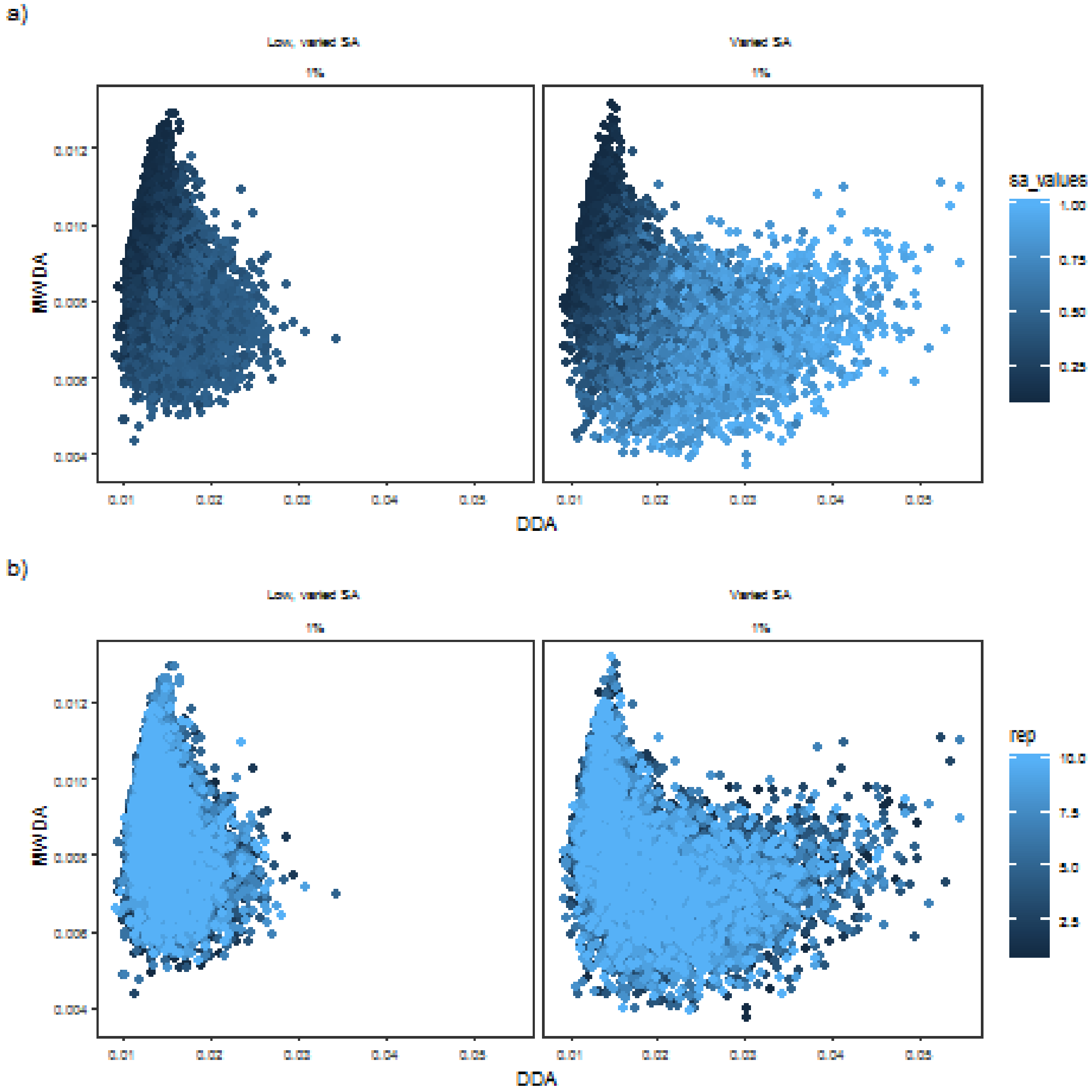

Figure AI.2 MWDA value plotted against DDA value for those scenarios resulting in negative correlations. In (a) colours represent the value of spatial autocorrelation in the individual cell, from 0 (low spatial autocorrelation) to 1 (high spatial autocorrelation). In (b) colours represent the replicate from which the data are shown.

In all cases, low values of spatial autocorrelation result in high MWDA and low DDA variance; high values of spatial autocorrelation result in low MWDA and high DDA This is what is driving the negative correlation. Within spatial autocorrelation values, we still see positive correlation between replicates, these correlations are greater for lower values of spatial autocorrelation. Within replicate, this manifests as negative correlation (leading to an overall negative correlation when taken as the mean of all replicates).

Question 2: Identifying the scale-of-effect

In order to examine whether we can correctly identify the scale of effect when comparing several correlated measures calculated using different window sizes, we use our MW measures to generate an ecological response variable for every window size in each coarse grid cell in each replicate landscape and all spatial autocorrelation scenarios. We calculate this response variable as $y_w = MW_w + \epsilon$ where y_w is the response variable for a given window size w, MW_w is the moving window calculated measure for window size w and ϵ $N(0, \sigma)$. We use three levels of ϵ:

- low ϵ: first percentile of the MWDA measure value within each spatial autocorrelation and window combination. This represents data with minimal noise.
- moderate ϵ: tenth percentile of the MWDA measure value within each spatial autocorrelation and window combination. This represents data with a moderate amount of noise.
- high ϵ: median of the MWDA measure value within each spatial autocorrelation and window combination. This represents data with a large amount of noise.

For each y_w we fit a univariate linear model with each MW_w as the covariate and use R^2 and AIC to examine whether the correct window size (and thus, scale of effect) is identified.

For the models fit for each of the y_w and MW_w combinations, we calculate the number of replicates where the best fitting model was the correct one.

```
window_y = unique(df$window)

noise_level = tibble(noise_level = c("Low noise", "Moderate noise", "High noise"),
                     noise_value = c(0.1, 0.25, 0.5))

df_narrow <- df %>%
  select(rep, ls_id, sa_scenario, window, cont_mwm, cat_mwm) %>%
  gather(measure, value, -rep, -ls_id, -sa_scenario, -window)

y_vals <- df_narrow %>%
  crossing(noise_level) %>%
  group_by(sa_scenario, window, measure) %>%
  mutate(sigma = noise_value * mean(value),
         eps = map_dbl(sigma, function(x) rnorm(1, 0, x)),
         y = value + eps) %>%
  ungroup() %>%
  select(rep, sa_scenario, ls_id, window_y = window, noise_level, measure, y)

mod_df <- df_narrow %>%
  rename(window_x = window) %>%
  crossing(window_y) %>%
  inner_join(y_vals) %>%
  group_by(rep, sa_scenario, noise_level, measure, window_x, window_y) %>%
  nest() %>%
  mutate(mod = map(data, function(x) lm(y ~ value, data = x)),
         mod_glance = map(mod, glance),
         mod_r2 = map_dbl(mod_glance, function(x) x$r.squared),
         mod_aic = map_dbl(mod_glance, function(x) x$AIC))

correct_df <- mod_df %>%
  select(rep, sa_scenario, window_x, window_y, noise_level, measure, mod_r2, mod_aic) %>%
  gather(key, value, -rep, -sa_scenario, -window_x, -window_y, -noise_level, -measure) %>%
  mutate(value = case_when(key == "mod_r2" ~ 1 - value, TRUE ~ value),
         ls_type = factor(measure, labels = c("Categorical", "Continuous")),
```

```
         fit_measure = factor(key,
                              labels = c("AIC", expression(R^{2}))),
         noise_level = factor(noise_level,
                             levels = c("Low noise", "Moderate noise", "High noise"))) %>%
  group_by(rep, sa_scenario, window_y, noise_level, ls_type, fit_measure) %>%
  slice(which.min(value)) %>%
  mutate(pass = window_x == window_y) %>%
  group_by(sa_scenario, window_y, noise_level, ls_type, fit_measure) %>%
  summarise(pass = sum(pass)) %>%
  ungroup()
```

```
ggplot(correct_df %>% filter(fit_measure == "AIC"),
       aes(x = window_y, y = sa_scenario, fill = pass)) +
  geom_tile() +
  scale_fill_viridis_c("% correct") +
  labs(x = "Window % of landscape", y = "") +
  facet_grid(noise_level ~ ls_type)
```

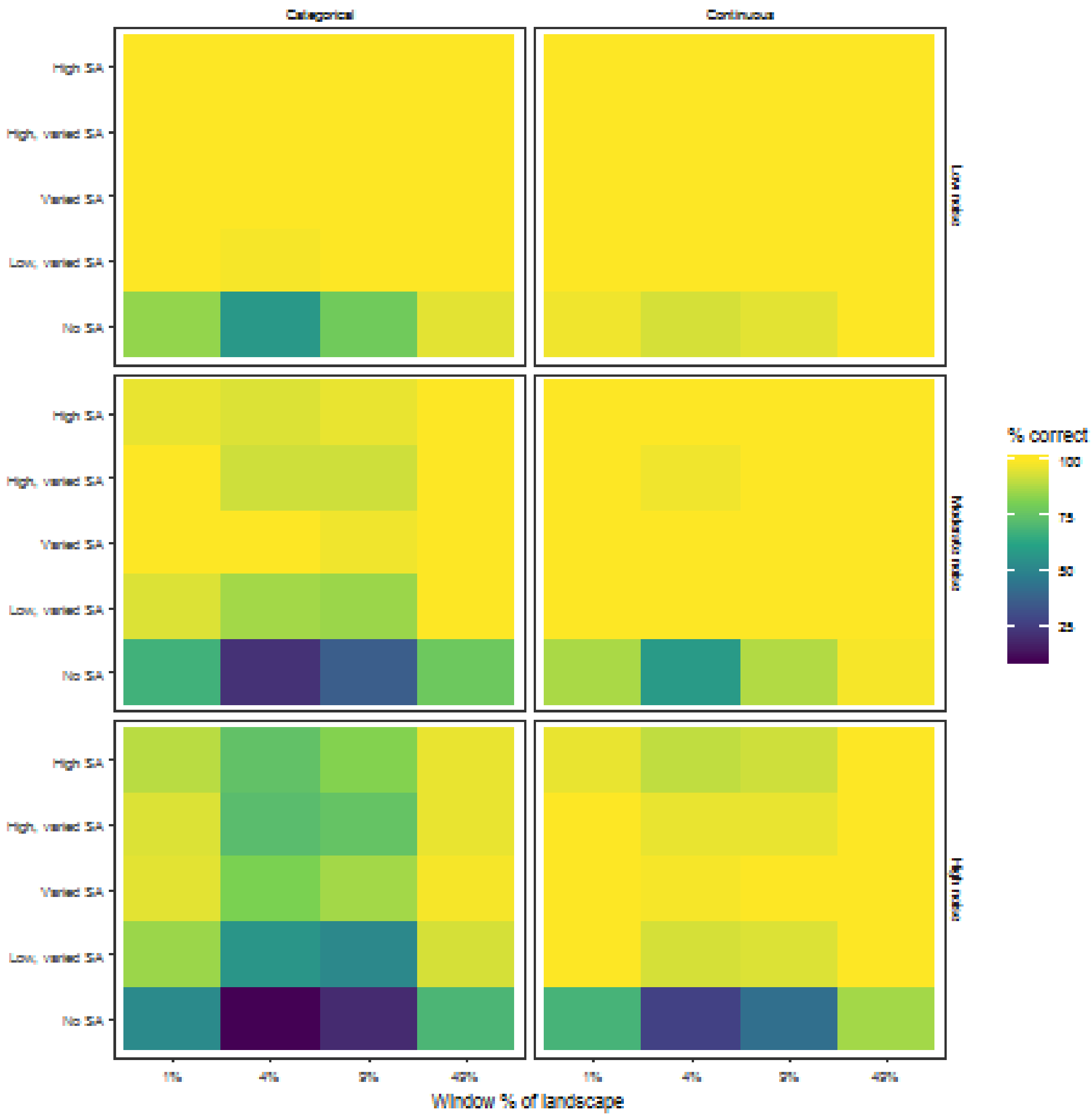

Figure AI.3 (a) correlation plot from above; (b) Percentage of replicates where the correct scale-of-effect was identified across the five landscape scenarios, four scales-of-effect and three noise levels.

```
correct_df %>% filter(fit_measure == "AIC") %>%
  group_by(ls_type, noise_level) %>%
  summarise_at(.vars = "pass", .funs = c("mean", "min", "max")) %>%
  kable()
```

ls_type	noise_level	mean	min	max
Categorical	Low noise	95.85	58	100
Categorical	Moderate noise	87.30	22	100
Categorical	High noise	74.60	10	99
Continuous	Low noise	99.40	94	100
Continuous	Moderate noise	96.65	59	100

ls_type	noise_level	mean	min	max
Continuous	High noise	89.45	27	100

Table AI.1 Mean, minimum and maximum percentage of replicates where the correct scale-of-effect was identified across the five landscape scenarios, four scales-of-effect and three noise levels.

```
correct_df %>% filter(fit_measure == "AIC") %>%
  group_by(ls_type, noise_level) %>%
  slice(which.min(pass)) %>%
  kable()
```

sa_scenario	window_y	noise_level	ls_type	fit_measure	pass
No SA	4%	Low noise	Categorical	AIC	58
No SA	4%	Moderate noise	Categorical	AIC	22
No SA	4%	High noise	Categorical	AIC	10
No SA	4%	Low noise	Continuous	AIC	94
No SA	4%	Moderate noise	Continuous	AIC	59
No SA	4%	High noise	Continuous	AIC	27

Table AI.2 Minimum number of replicates where correct scale-of-effect was identified across the five landscape scenarios and three noise levels.

- Whether judged by R^2 or AIC, we are able to identify the correct scale of effect in low noise datasets most of the time
- In moderate and high noise datasets, we are best able to identify the correct scale of effect when it is small or large, and the data is continuous.
- Overall, we were least successful in identifying the scale of effect in the No spatial autocorrelation scenario

It's also useful to understand the general fit of the models at each spatial autocorrelation and window size combination.

```
r2_df <- mod_df %>%
  select(measure, noise_level, sa_scenario, window_x, window_y, mod_r2) %>%
  filter(window_x == window_y) %>%
  mutate(ls_type = factor(measure, labels = c("Categorical", "Continuous")),
         noise_level = factor(noise_level,
                              levels = c("Low noise", "Moderate noise", "High noise"))) %>%
  group_by(ls_type, noise_level, sa_scenario, window_x) %>%
  summarise(mean_r2 = round(mean(mod_r2), 2))

ggplot(r2_df, aes(x = window_x, y = sa_scenario, fill = mean_r2)) +
  geom_tile() +
  scale_fill_viridis_c(expression(R^2)) +
  labs(x = "Window % of landscape", y = "") +
  facet_grid(noise_level ~ ls_type)
```

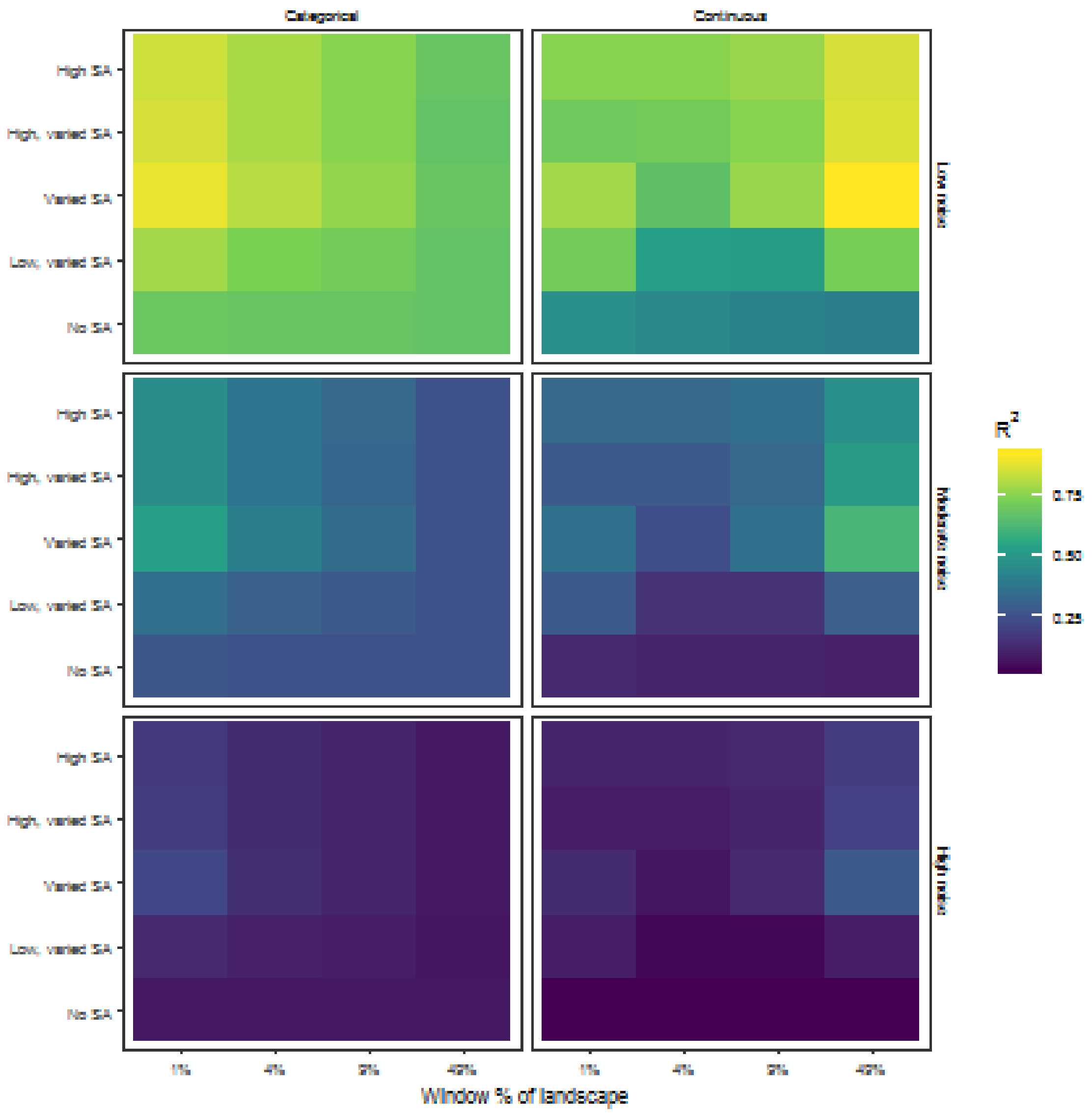

Figure AI.4 Mean R^2 values from the models for the correct scale-of-effect across the five landscape scenarios, four window sizes and three noise levels.

```
r2_df %>% group_by(ls_type, noise_level) %>%
  summarise_at(.vars = "mean_r2", .funs = c("min", "mean", "max")) %>%
  kable()
```

ls_type	noise_level	min	mean	max
Categorical	Low noise	0.67	0.7430	0.88
Categorical	Moderate noise	0.25	0.3275	0.53
Categorical	High noise	0.07	0.1120	0.22
Continuous	Low noise	0.41	0.6740	0.91
Continuous	Moderate noise	0.10	0.2885	0.61
Continuous	High noise	0.03	0.0995	0.28

Table AI.3 Mean, minimum and maximum R^2 values from the models for the correct scale-of-effect across

the five landscape scenarios, four window sizes and three noise levels.

Session Info

```
session <- devtools::session_info()
session[[1]]
```

```
##  setting  value
##  version  R version 3.5.1 (2018-07-02)
##  os       Windows 10 x64
##  system   x86_64, mingw32
##  ui       RTerm
##  language (EN)
##  collate  English_United Kingdom.1252
##  ctype    English_United Kingdom.1252
##  tz       Europe/London
##  date     2019-02-15
```

```
session[[2]] %>% select(package, loadedversion, source) %>% kable
```

	package	loadedversion	source
assertthat	assertthat	0.2.0	CRAN (R 3.5.0)
backports	backports	1.1.2	CRAN (R 3.5.0)
base64enc	base64enc	0.1-3	CRAN (R 3.5.0)
bindr	bindr	0.1.1	CRAN (R 3.5.0)
bindrcpp	bindrcpp	0.2.2	CRAN (R 3.5.0)
broom	broom	0.5.0	CRAN (R 3.5.1)
callr	callr	3.0.0	CRAN (R 3.5.1)
cellranger	cellranger	1.1.0	CRAN (R 3.5.0)
cli	cli	1.0.1	CRAN (R 3.5.1)
codetools	codetools	0.2-15	CRAN (R 3.5.1)
colorspace	colorspace	1.4-0	CRAN (R 3.5.2)
cowplot	cowplot	0.9.3	CRAN (R 3.5.1)
crayon	crayon	1.3.4	CRAN (R 3.5.0)
debugme	debugme	1.1.0	CRAN (R 3.5.0)
desc	desc	1.2.0	CRAN (R 3.5.0)
devtools	devtools	2.0.0	CRAN (R 3.5.1)
digest	digest	0.6.18	CRAN (R 3.5.1)
dplyr	dplyr	0.7.7	CRAN (R 3.5.1)
evaluate	evaluate	0.12	CRAN (R 3.5.1)
extrafont	extrafont	0.17	CRAN (R 3.5.0)
extrafontdb	extrafontdb	1.0	CRAN (R 3.5.0)
forcats	forcats	0.3.0	CRAN (R 3.5.0)
fs	fs	1.2.6	CRAN (R 3.5.2)
ggplot2	ggplot2	3.1.0	CRAN (R 3.5.1)
glue	glue	1.3.0.9000	Github (tidyverse/glue@3f7012c)
grainchanger	grainchanger	0.0.0.9000	local
gtable	gtable	0.2.0	CRAN (R 3.5.0)
haven	haven	1.1.2	CRAN (R 3.5.1)
highr	highr	0.7	CRAN (R 3.5.1)
hms	hms	0.4.2	CRAN (R 3.5.0)
htmltools	htmltools	0.3.6	CRAN (R 3.5.0)
httr	httr	1.3.1	CRAN (R 3.5.0)

	package	loadedversion	source
jsonlite	jsonlite	1.6	CRAN (R 3.5.2)
knitr	knitr	1.21	CRAN (R 3.5.2)
labeling	labeling	0.3	CRAN (R 3.5.0)
landscapetools	landscapetools	0.4.0	CRAN (R 3.5.1)
lattice	lattice	0.20-35	CRAN (R 3.5.1)
lazyeval	lazyeval	0.2.1	CRAN (R 3.5.0)
lubridate	lubridate	1.7.4	CRAN (R 3.5.0)
magrittr	magrittr	1.5	CRAN (R 3.5.0)
memoise	memoise	1.1.0	CRAN (R 3.5.0)
modelr	modelr	0.1.2	CRAN (R 3.5.0)
munsell	munsell	0.5.0	CRAN (R 3.5.1)
nlme	nlme	3.1-137	CRAN (R 3.5.1)
NLMR	NLMR	0.3.2	CRAN (R 3.5.1)
patchwork	patchwork	0.0.1	Github (thomasp85/patchwork@7fb35b1)
pillar	pillar	1.3.1	CRAN (R 3.5.2)
pkgbuild	pkgbuild	1.0.2	CRAN (R 3.5.1)
pkgconfig	pkgconfig	2.0.2	CRAN (R 3.5.1)
pkgload	pkgload	1.0.2	CRAN (R 3.5.2)
plyr	plyr	1.8.4	CRAN (R 3.5.0)
prettyunits	prettyunits	1.0.2	CRAN (R 3.5.0)
processx	processx	3.2.0	CRAN (R 3.5.1)
ps	ps	1.2.0	CRAN (R 3.5.1)
purrr	purrr	0.2.5	CRAN (R 3.5.1)
R6	R6	2.3.0	CRAN (R 3.5.1)
raster	raster	2.8-6	Github (rspatial/raster@589de57)
RColorBrewer	RColorBrewer	1.1-2	CRAN (R 3.5.0)
Rcpp	Rcpp	1.0.0	CRAN (R 3.5.2)
readr	readr	1.1.1	CRAN (R 3.5.0)
readxl	readxl	1.1.0	CRAN (R 3.5.0)
remotes	remotes	2.0.1	CRAN (R 3.5.1)
reshape2	reshape2	1.4.3	CRAN (R 3.5.0)
rlang	rlang	0.3.0.1	CRAN (R 3.5.1)
rmarkdown	rmarkdown	1.11	CRAN (R 3.5.2)
rprojroot	rprojroot	1.3-2	CRAN (R 3.5.0)
rstudioapi	rstudioapi	0.8	CRAN (R 3.5.1)
Rttf2pt1	Rttf2pt1	1.3.7	CRAN (R 3.5.0)
rvest	rvest	0.3.2	CRAN (R 3.5.0)
scales	scales	1.0.0	CRAN (R 3.5.1)
sessioninfo	sessioninfo	1.1.0	CRAN (R 3.5.1)
sp	sp	1.3-1	CRAN (R 3.5.1)
stringi	stringi	1.2.4	CRAN (R 3.5.1)
stringr	stringr	1.3.1	CRAN (R 3.5.0)
testthat	testthat	2.0.1	CRAN (R 3.5.1)
tibble	tibble	2.0.0	CRAN (R 3.5.1)
tidyr	tidyr	0.8.2	CRAN (R 3.5.1)
tidyselect	tidyselect	0.2.5	CRAN (R 3.5.1)
tidyverse	tidyverse	1.2.1	CRAN (R 3.5.0)
usethis	usethis	1.4.0	CRAN (R 3.5.2)
viridisLite	viridisLite	0.3.0	CRAN (R 3.5.0)
withr	withr	2.1.2	CRAN (R 3.5.1)
xfun	xfun	0.4	CRAN (R 3.5.1)
xml2	xml2	1.2.0	CRAN (R 3.5.0)

	package	loadedversion	source
yaml	yaml	2.2.0	CRAN (R 3.5.1)
