# Supplementary material for: Incorporating fine‐scale environmental heterogeneity into broad‐extent models
Source: Methods Ecol Evol. 2019 Apr 8;10(6):767–78. doi: 10.1111/2041-210X.13177 (PMC6582547; doi:10.1111/2041-210X.13177)
Supplement: Supplementary file 2 [file MEE3-10-767-s002.pdf]

Appendix II: *Garrulus glandarius* case study

Required packages

In the first step, we load all of the required packages. Note that grainchanger (the package developed for this paper) can be installed using:

```
devtools::install_github("laurajanegraham/grainchanger")
```

Other options in this section set the order and labels for the variables for plotting and tables.

```r
library(jtools)
library(perturb)
library(car)
library(grainchanger)
library(raster)
library(sf)
library(knitr)
library(e1071) # optimising transformations (skewness function)
library(broom)
library(stringr)
library(DHARMa)
library(tidyverse)
library(corrr)
library(cowplot)

# define logit and inv logit functions
logit <- function(value, eps) {
  log((value + eps)/(1 - value + eps))
}

inv.logit <- function(value, eps) {
  eps <- (1-2*eps)
  (eps*(1+exp(value))+(exp(value)-1))/(2*eps*(1+exp(value)))
}

# set up plotting options
theme_set(theme_bw(base_size = 8) + theme(strip.background = element_blank(),
                              panel.grid.major = element_blank(),
                              panel.grid.minor = element_blank()))

# set up some options
# for exploratory plotting
varorder <- c("index10",
              "winshannon50",
              "winshannon100",
              "winshannon500",
              "winshannon1000",
              "winshannon1500",
              "winshannon3500",
              "lsshannon",
              "habitat",
              "urban",
```

```
              "bio1")

varlabel <- c("Abundance (2010 Atlas)",
              "MWDA Shannon\n(50 m)",
              "MWDA Shannon\n(100 m)",
              "MWDA Shannon\n(500 m)",
              "MWDA Shannon\n(1 km)",
              "MWDA Shannon\n(1.5 km)",
              "MWDA Shannon\n(3.5 km)",
              "DDA Shannon",
              "Forest %",
              "Urban %",
              "Temperature")

# for initial tables from models
coefforder <- c("(Intercept)",
                "winshannon50",
                "winshannon100",
                "winshannon500",
                "winshannon1000",
                "winshannon1500",
                "winshannon3500",
                "lsshannon",
                "habitat",
                "winshannon:bio1",
                "lsshannon:bio1",
                "urban",
                "bio1")

coefflabel <- c("Intercept",
                "MWDA Shannon\n(50 m)",
                "MWDA Shannon\n(100 m)",
                "MWDA Shannon\n(500 m)",
                "MWDA Shannon\n(1 km)",
                "MWDA Shannon\n(1.5 km)",
                "MWDA Shannon\n(3.5 km)",
                "DDA Shannon",
                "Forest %",
                "MWDA Shannon : Temperature",
                "DDA Shannon : Temperature",
                "Urban %",
                "Temperature")
```

Data

Note that file paths to data sources are hard coded. These will need updating to match folder structure. A search for `~/` in the document will find these.

Biological data

First we need to load in and spatialise the Jay (*Garrulus glandarius*) data. These were provided by Simon Gillings at the BTO. The data includes the relative abundance indices for the 1990 Atlas (index90) and for the 2010 Atlas (index10). We are using the 2010 Atlas data.

```
jay_sp <- read_sf("~/DATA/ADMINISTRATIVE/bng/10km_grid_region.shp") %>%
  rename(grid = TILE_NAME) %>%
  inner_join(read_csv("~/DATA/BIOLOGICAL/bto_jays/extract.csv") %>%
               rename(grid = tenkm)) %>%
  select(index10, geometry) %>%
  filter(!is.na(geometry))
```

Environmental data

The data for which we want to use the upscaling approach on is Land Cover Map 2007, which is the closest match to the 2010 relative abundance index for jays. We will use the moving window to upscale diversity of the two used habitats: Broadleaf and Coniferous forest (LCM codes 1, 2). Eurasian jays use a combination of these habitats: broadleaf for foraging, coniferous for nesting. We will calculate Shannon Diversity on just these two habitats to create a measure of the landscape structure used by jays. We will calculate Shannon diversity using the moving window approach at the 1km scale and without the moving window at the 10km scale. As covariates, we will calculate forest (habitat) cover percentage, urban land cover percentage and from Worldclim mean annual temperature (bio1).

```
lcm <- raster("~/DATA/LULC/lcm2007/lcm2007_25m_gb.tif")
forest <- c(1, 2) # these are the two forest classes
urban <- c(22, 23)

# allow for multicore processing
plan(multiprocess)

# shannon via moving window
strt <- Sys.time()
jay_sp$winshannon50 <- grainchanger::winmove_agg(g = jay_sp, dat = lcm, d = 50,
                                              type = "rectangle", fun = "shei",
                                              lc_class = forest)
runtime50 <- difftime(Sys.time(), strt, units = "mins")

# shannon via moving window
strt <- Sys.time()
jay_sp$winshannon100 <- grainchanger::winmove_agg(g = jay_sp, dat = lcm, d = 100,
                                              type = "rectangle", fun = "shei",
                                              lc_class = forest)
runtime100 <- difftime(Sys.time(), strt, units = "mins")

strt <- Sys.time()
jay_sp$winshannon500 <- grainchanger::winmove_agg(g = jay_sp, dat = lcm, d = 500,
                                              type = "rectangle", fun = "shei",
                                              lc_class = forest)
runtime500 <- difftime(Sys.time(), strt, units = "mins")

strt <- Sys.time()
jay_sp$winshannon1000 <- grainchanger::winmove_agg(g = jay_sp, dat = lcm, d = 1000,
                                              type = "rectangle", fun = "shei",
                                              lc_class = forest)
runtime1000 <- difftime(Sys.time(), strt, units = "mins")

strt <- Sys.time()
jay_sp$winshannon1500 <- grainchanger::winmove_agg(g = jay_sp, dat = lcm, d = 1500,
                                              type = "rectangle", fun = "shei",
```

```
                                                     lc_class = forest)
runtime1500 <- difftime(Sys.time(), strt, units = "mins")

strt <- Sys.time()
jay_sp$winshannon3500 <- grainchanger::winmove_agg(g = jay_sp, dat = lcm, d = 3500,
                                                     type = "rectangle", fun = "shei",
                                                     lc_class = forest)
runtime3500 <- difftime(Sys.time(), strt, units = "mins")

runtime <- tibble(d = c(50, 100, 500, 1000, 1500, 3500),
                  runtime = c(runtime50,
                              runtime100,
                              runtime500,
                              runtime1000,
                              runtime1500,
                              runtime3500))
save(runtime, file = "results/jays_runtime.Rda")

# shannon without moving window
jay_sp$lsshannon <- grainchanger::nomove_agg(g = jay_sp, dat = lcm,
                                fun = "diversity", lc_class = forest)

# other measures from lcm
jay_sp$habitat <- grainchanger::nomove_agg(g = jay_sp,
                                           dat = lcm,
                                           fun = "prop",
                                           lc_class = forest)
jay_sp$urban <- grainchanger::nomove_agg(g = jay_sp,
                                         dat = lcm,
                                         fun = "prop",
                                         lc_class = urban)

# Bioclimatic variables from worldclim
wc_bio <- getData('worldclim', var = 'bio', path = '~/DATA/CLIMATE/worldclim/', res=5)
jay_pts <- st_centroid(jay_sp) %>% st_transform(proj4string(wc_bio))
jay_sp <- jay_sp %>% bind_cols(raster::extract(wc_bio, jay_pts) %>% as.tibble)

save(jay_sp, file="results/jays_covariates.Rda")
```

Running times to aggregate 25m resolution land cover data to 10km for the 4 different window sizes:

```
load("results/jays_runtime.Rda")
runtime %>% kable(col.names = c("Window size", "Runtime"), digits = 0)
```

Window size	Runtime
50	3 mins
100	3 mins
500	10 mins
1000	29 mins
1500	64 mins
3500	490 mins

Exploration and transformation

We have removed cells with 0 abundance because we are only really interested in predicting abundance presuming the species is present. Alternatively we could use all data and a hurdle model, however we chose to take the simpler approach.

```
load("results/jays_covariates.Rda")
jay_sp <- jay_sp %>%
  filter(index10 != 0) %>%
  na.omit %>%
  select(varorder) %>%
  mutate(bio1 = bio1/10)
# /10 is due to way that worldclim stores the temperature data

jay_df <- jay_sp %>%
  as_tibble %>%
  select(-geometry)
```

What do the variables look like spatially?

```
gb_outline <- st_read("~/DATA/ADMINISTRATIVE/gb_shapefile/GBR_adm1.shp",
                      quiet = TRUE) %>%
  filter(NAME_1 != "Northern Ireland") %>%
  st_crop(xmin = -8, ymin = 49.865417, xmax = 1.764168, ymax = 61.527084)

jay_narrow <- jay_sp %>%
  mutate_at(.vars = vars(-index10, -geometry), .funs = funs(scale)) %>%
  gather(variable, value, -geometry) %>%
  mutate(facet = "",
         fvariable = factor(variable, levels = varorder, labels = varlabel))

jay_plot <- ggplot(jay_narrow %>% filter(variable == "index10")) +
  geom_sf(data = gb_outline, fill = "grey", colour = NA) +
  geom_sf(aes(fill = value), colour = NA) +
  coord_sf(crs = st_crs(jay_narrow), datum = NA) +
  scale_fill_viridis_c(name = "", option = "magma") +
  facet_wrap(~facet) +
  theme(axis.text = element_blank(), axis.line = element_blank(),
        axis.ticks = element_blank(),
        legend.position = "bottom", legend.title.align = 0.5,
        legend.key.height=unit(6,"points"), legend.key.width = unit(1.5, "line"),
        panel.border = element_blank())

cov_plot <- ggplot(jay_narrow %>%
                     filter(variable %in% c("winshannon100",
                                            "habitat",
                                            "urban",
                                            "bio1"))) +
  geom_sf(data = gb_outline, fill = "grey", colour = NA) +
  geom_sf(aes(fill = value), colour = NA) +
  coord_sf(crs = st_crs(jay_narrow), datum = NA) +
  scale_fill_viridis_c(name = "") + facet_wrap(~fvariable) +
  theme(axis.text = element_blank(), axis.line = element_blank(),
        axis.ticks = element_blank(),
        legend.position = "bottom", legend.title.align = 0.5,
```

```r
        legend.key.height=unit(6,"points"), legend.key.width = unit(2, "line"),
        panel.border = element_blank())

plot_grid(jay_plot,
          cov_plot,
          labels = c("a)", "b)"),
          label_size = 7,
          rel_widths = c(1, 1.5))
```

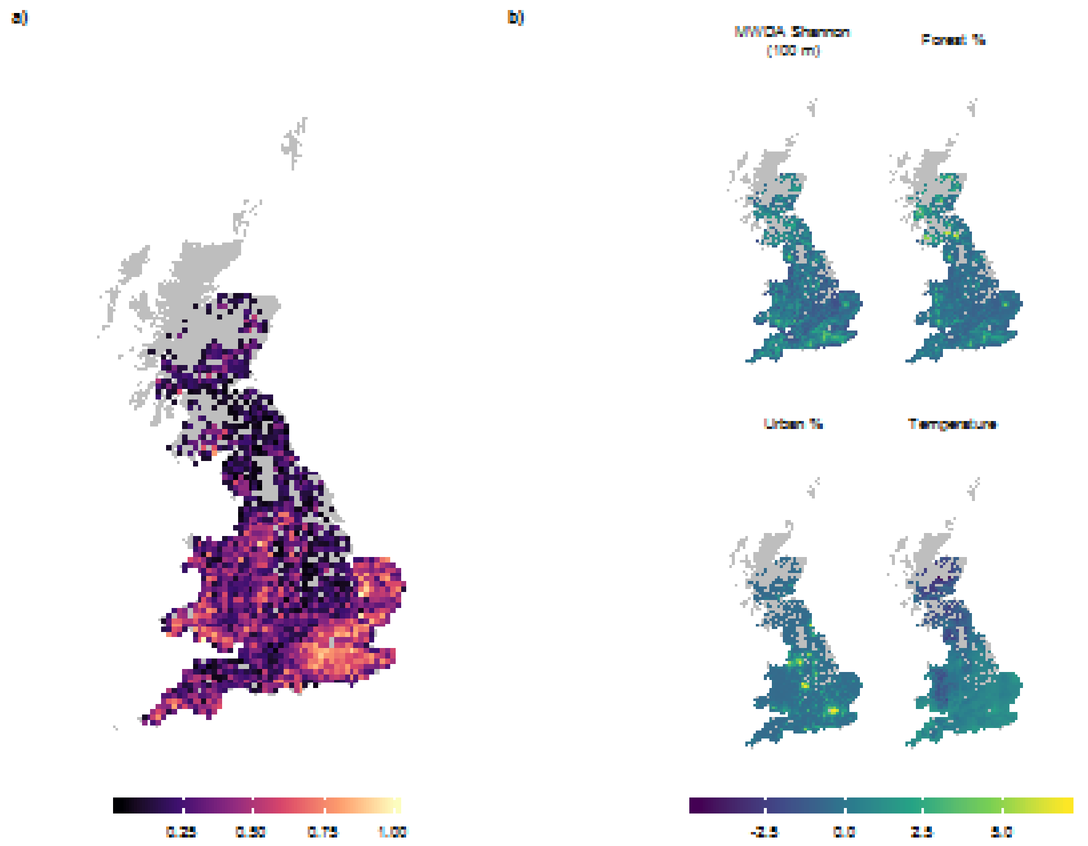

Figure AII.1 A case study of the effect of habitat structure on Garrulus glandarius abundance. Study area showing the spatial distribution of (a) relative abundance index for G. glandarius and (b) scaled covariates (mean = 0; standard deviation = 1).

How are the variables distributed and where are the correlations?

```r
ggplot(jay_narrow, aes(x = value)) +
      geom_histogram() +
      facet_wrap(~ variable, scales = "free")
```

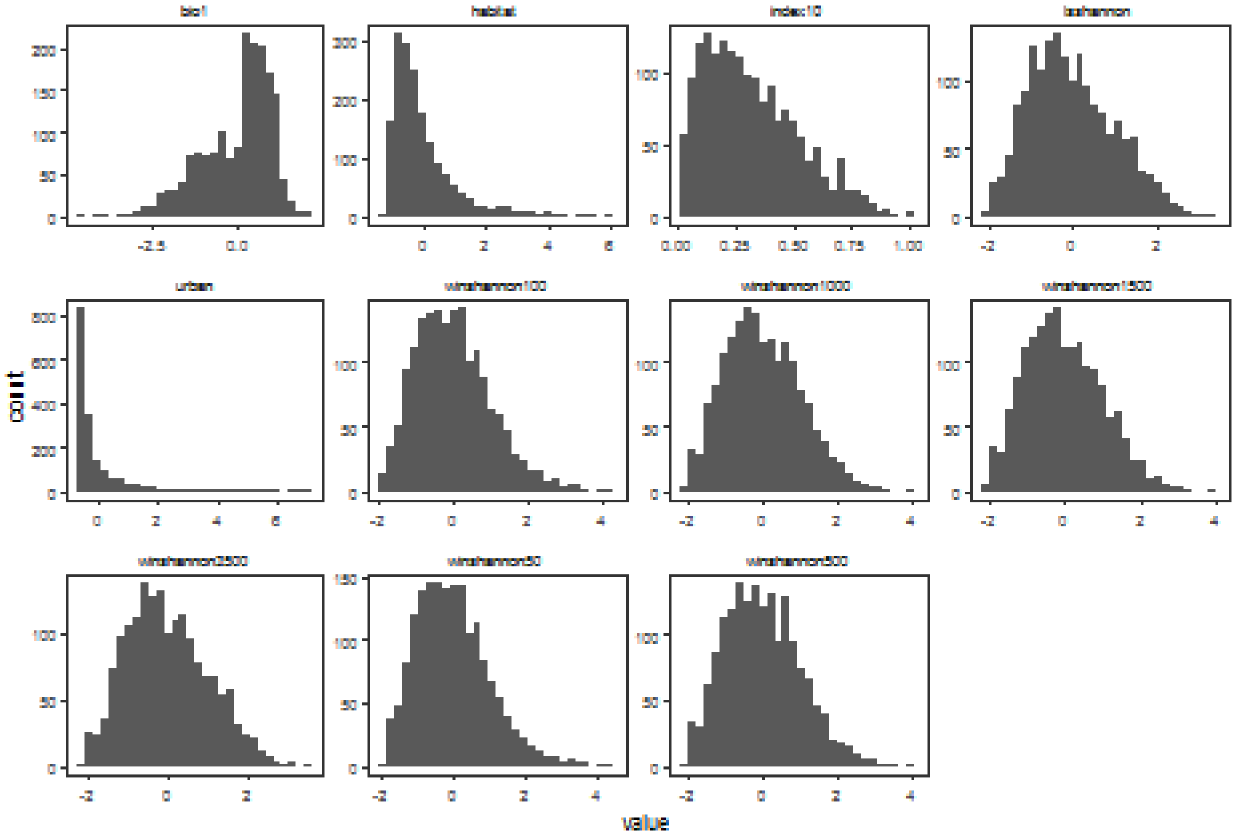

Figure AII.2 Distributions of *Garrulus glandarius* relative abundance and covariates.

Right skew to habitat percentage, urban percentage and precipitation, and left skew to temperature.

```
corrs <- jay_df %>%
  correlate(method = "spearman", quiet = TRUE) %>%
  shave

rplot(corrs, shape = 15, print_cor = TRUE) +
  theme(axis.text.x = element_text(angle = 90, hjust = 1))
```

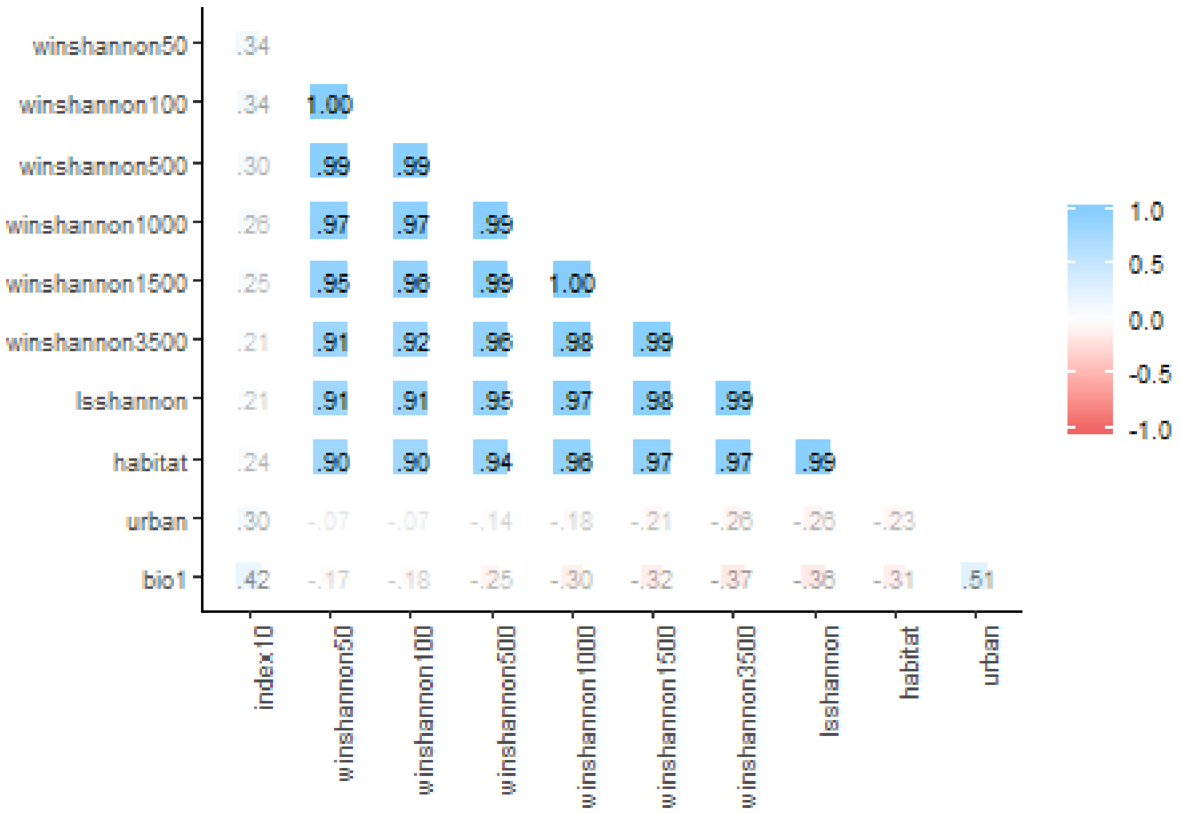

Figure AII.3 Pairwise Spearman correlations between *Garrulus glandarius* relative abundance and covariates.

MWDA Shannon a stronger correlate of relative abundance than DDA Shannon, smaller the window, the stronger the correlation. Although all Shannon measures correlate with the amount of habitat, MWDA Shannon (50m & 100m) is least correlated with this (still very high).

Despite skew in the data, we have not transformed these variables because model assumptions are met without doing so (ease of interpretation). Because the abundance index is proportional, we transform this using a logit transform with the smallest non-zero value added to the numerator and denominator due to presence of 1s in data:

```r
# smallest relative abundance
eps <- min(jay_df$index10)
jay_df_t <- mutate(jay_df,
                   index10logit = logit(index10, eps))

jay_narrow_t <- gather(jay_df_t, variable, value) %>%
  mutate(variable = factor(variable,
         levels = varorder, labels = varlabel))

# get mean and sd values from the transformed data for back-scaling
means <- jay_df_t %>%
  summarise_all(funs(mean, sd)) %>%
  gather() %>%
  separate(key, into=c("covariate", "measure")) %>%
  spread(measure, value)
```

The abundance score for two cells was equal to 1, so the smallest non-zero percentage response (0.03) was

added to the index before applying the logit function to avoid divide by zero issues.

We scaled the data (mean = 0, SD = 1) to make the covariates comparable due to the wide range in measurement scales.

```
scale_this <- function(x) as.vector(scale(x))

jay_df_t <- mutate_at(jay_df_t,
                      .vars = vars(-index10logit, -index10),
                      .funs = funs(scale_this))
```

Statistical model

Our sample size is n = 1719.

Our model contains the following variables: Shannon measure, Forest %, Urban % and Temperature. We are also including the interaction term between Temperature and each Shannon measure. We fit this model for all 4 window sizes of MWDA Shannon and SDA Shannon.

```
jay_mod <- jay_df_t %>%
  gather(window_size, shannon, -index10, -index10logit, -habitat, -urban, -bio1) %>%
  group_by(window_size) %>%
  nest() %>%
  mutate(mod = map(data, function(x) lm(index10logit ~ shannon + habitat + urban +
                                           bio1 + bio1:shannon,
                                         data = x, na.action = na.fail)),
         mod_res = map(mod, function(x) bind_cols(tidy(x), confint_tidy(x))),
         mod_glance = map(mod, glance),
         mod_check = map(mod, function(x) {
           tibble(resids = x$residuals, fitted = x$fitted)
         }))

mod_comp <- jay_mod %>%
  select(window_size, mod_glance) %>%
  unnest() %>%
  select(window_size, AIC, BIC) %>%
  arrange(AIC)

best_mod <- mod_comp %>%
  slice(1) %>%
  pull(window_size)

mod_comp %>%
  kable(digits = 3)
```

window_size	AIC	BIC
winshannon100	3975.011	4013.158
winshannon50	3976.366	4014.512
winshannon500	3977.424	4015.571
winshannon1000	3984.681	4022.828
winshannon1500	3990.993	4029.140
winshannon3500	4001.924	4040.070
lsshannon	4005.014	4043.160

Table AII.1 Results of model comparison

The best fitting model (judged by AIC and BIC is that with the 100m window).

Model validation

```
jay_df_best <- jay_mod %>%
  filter(window_size == best_mod) %>%
  select(data, mod_check) %>%
  unnest()

# fitted values on the data scale
fitted <- inv.logit(jay_df_best$fitted, eps)

plot_grid(
  ggplot(jay_df_best, aes(x = fitted, y = resids)) +
    geom_point(size = 0.1) + geom_hline(yintercept = 0),
  ggplot(jay_df_best, aes(x = shannon, y = resids)) +
    geom_point(size = 0.1) + geom_hline(yintercept = 0) + xlab("MWDA Shannon (100m)"),
  ggplot(jay_df_best, aes(x = habitat, y = resids)) +
    geom_point(size = 0.1) + geom_hline(yintercept = 0) + xlab("Forest %"),
  ggplot(jay_df_best, aes(x = urban, y = resids)) +
    geom_point(size = 0.1) + geom_hline(yintercept = 0) + xlab("Urban %"),
  ggplot(jay_df_best, aes(x = bio1, y = resids)) +
    geom_point(size = 0.1) + geom_hline(yintercept = 0) + xlab("Temperature")
)
```

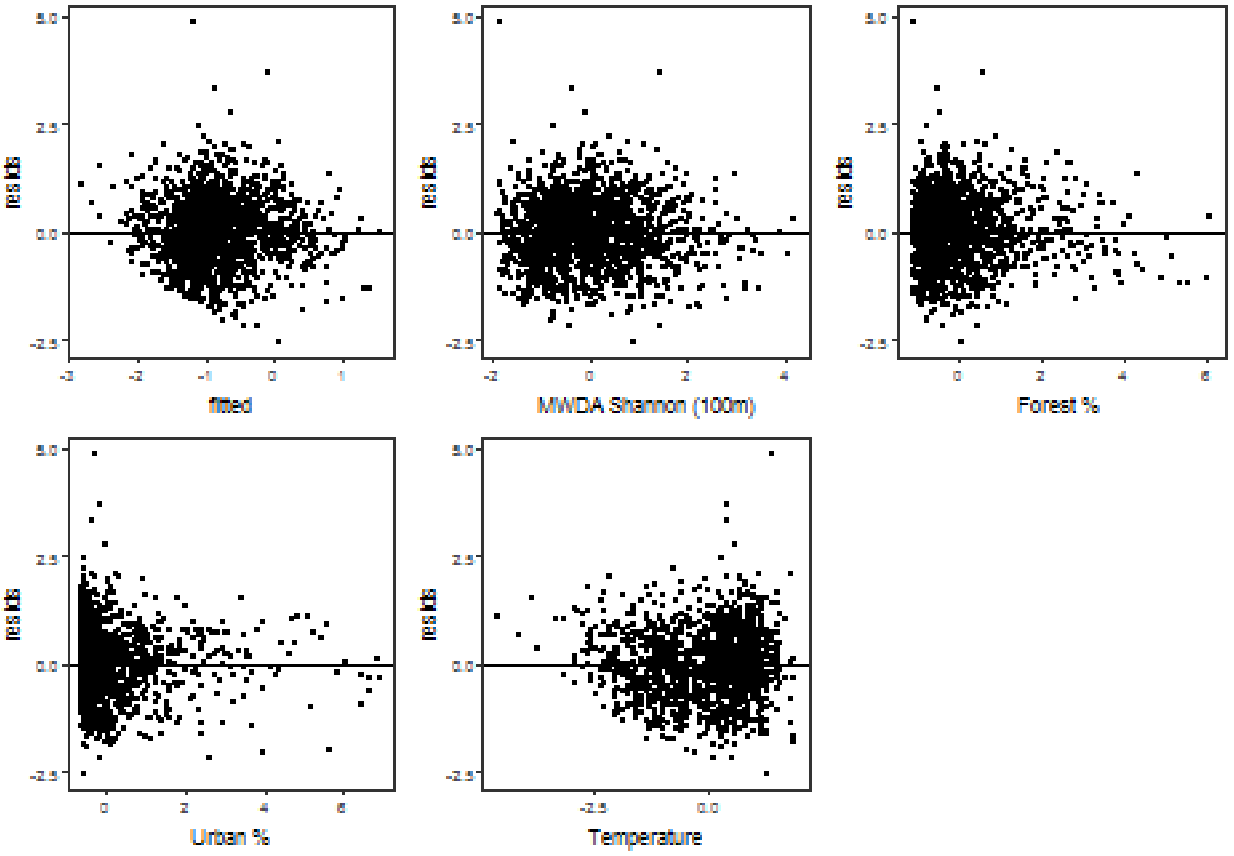

Figure AII.4 Residuals plotted against fitted values and all predictors.

There is some patterning in the residuals, but overall a reasonable model fit. The lowest fitted value is 0.03 and the highest is 0.84. Much better conformity to assumptions and range of predicted values than either when fit using untransformed response with either Gaussian or binomial distribution.

Final results and plots

Results of the best model

```
res_best <- jay_mod %>%
  filter(window_size == best_mod) %>%
  select(mod_res) %>%
  unnest()

r2_best <- jay_mod %>%
  filter(window_size == best_mod) %>%
  select(mod_glance) %>%
  unnest() %>%
  select(r.squared)

res_best %>%
  select(term, estimate, conf.low, conf.high) %>%
  kable(digits=3)
```

term	estimate	conf.low	conf.high
(Intercept)	-0.825	-0.862	-0.789
shannon	0.335	0.280	0.390
habitat	0.078	0.021	0.136
urban	0.129	0.091	0.168
bio1	0.427	0.385	0.468
shannon:bio1	0.090	0.052	0.128

Table AII.2 Results of linear regression for the best model.

The model explains 36.55% of the variance in jay abundance.

For those variables involved in an interaction, we plot the interaction plot; for those whose main effects we wish to interpret, we plot the effect plot.

```
jay_mod_best <- jay_mod %>%
  filter(window_size == best_mod) %>%
  select(mod)

jay_mod_best <- jay_mod_best[[1]][[1]]

means$covariate <- ifelse(means$covariate == best_mod, "shannon", means$covariate)

# need to predict from the model then convert to the original scales
pred_vals <- bind_rows(
  make_predictions(jay_mod_best, pred = "urban", interval = TRUE)[[1]] %>%
    select(index10logit, ymax, ymin, pred = urban) %>%
    mutate(covariate = "urban"),
  make_predictions(jay_mod_best, pred = "habitat", interval = TRUE)[[1]] %>%
```

```
    select(index10logit, ymax, ymin, pred = habitat) %>%
    mutate(covariate = "habitat"),
  make_predictions(jay_mod_best, pred = "bio1", modx = "shannon", interval = TRUE)[[1]] %>%
    select(index10logit, ymax, ymin, modx_group, pred = bio1) %>%
    mutate(covariate = "bio1", modx = "shannon"),
  make_predictions(jay_mod_best, pred = "shannon", modx = "bio1", interval = TRUE)[[1]] %>%
    select(index10logit, ymax, ymin, modx_group, pred = shannon) %>%
    mutate(covariate = "shannon", modx = "bio1")) %>%
  as.tibble %>%
  mutate_at(c("index10logit", "ymin", "ymax"), inv.logit, eps) %>%
  left_join(means) %>%
  left_join(means, by = c("modx" = "covariate"), suffix = c("_pred", "_modx")) %>%
  mutate(pred = pred*sd_pred + mean_pred,
         modx_value = case_when(modx_group == "- 1 SD" ~ mean_modx - sd_modx,
                                modx_group == "Mean" ~ mean_modx,
                                modx_group == "+ 1 SD" ~ mean_modx + sd_modx,
                                TRUE ~ 0),
         modx_label = factor(paste0(modx_group, " (", round(modx_value, 2), ")")),
         modx_label = fct_reorder(modx_label, modx_value))

# plot the results
shannon_plot <- ggplot(pred_vals %>% filter(covariate == "shannon"),
                     aes(x = pred, y = index10logit, group = modx_label)) +
  geom_line(aes(lty = modx_label)) +
  scale_linetype_manual(name = expression("Temperature (" * degree * "C)"),
                        values = c("dashed", "solid", "dotdash")) +
  scale_y_continuous(limits = c(0, 1), breaks = c(0, 0.25, 0.5, 0.75, 1)) +
  geom_ribbon(aes(ymin = ymin, ymax = ymax), alpha = 0.2) +
  theme(legend.position = c(0.25, 0.8), legend.key.height = unit(0.1, "line")) +
  labs(x = "MWDA Shannon (100 m)",
       y = expression(paste(italic("G. glandarius"),
                            " abundance index (" %+-% "95% CI)")))

bio1_plot <- ggplot(pred_vals %>% filter(covariate == "bio1"),
                     aes(x = pred, y = index10logit, group = modx_label)) +
  geom_line(aes(lty = modx_label)) +
  scale_linetype_manual(name = "MWDA Shannon (100 m)",
                        values = c("dashed", "solid", "dotdash")) +
  scale_y_continuous(limits = c(0, 1), breaks = c(0, 0.25, 0.5, 0.75, 1)) +
  geom_ribbon(aes(ymin = ymin, ymax = ymax), alpha = 0.2) +
  theme(legend.position = c(0.3, 0.8), legend.key.height = unit(0.1, "line")) +
  labs(x = expression("Temperature (" * degree * "C)"),
       y = "")

urban_plot <- ggplot(pred_vals %>% filter(covariate == "urban"),
                     aes(x = pred, y = index10logit)) +
  geom_line() +
  scale_y_continuous(limits = c(0, 1), breaks = c(0, 0.25, 0.5, 0.75, 1)) +
  geom_ribbon(aes(ymin = ymin, ymax = ymax), alpha = 0.2) +
  labs(x = "Urban %",
       y = expression(paste(italic("G. glandarius"),
                            " abundance index (" %+-% "95% CI)")))
```

```
habitat_plot <- ggplot(pred_vals %>% filter(covariate == "habitat"),
                       aes(x = pred, y = index10logit)) +
  geom_line() +
  scale_y_continuous(limits = c(0, 1), breaks = c(0, 0.25, 0.5, 0.75, 1)) +
  geom_ribbon(aes(ymin = ymin, ymax = ymax), alpha = 0.2) +
  labs(x = "Forest %",
       y = "")

plot_grid(shannon_plot, bio1_plot, urban_plot, habitat_plot, nrow = 2)
```

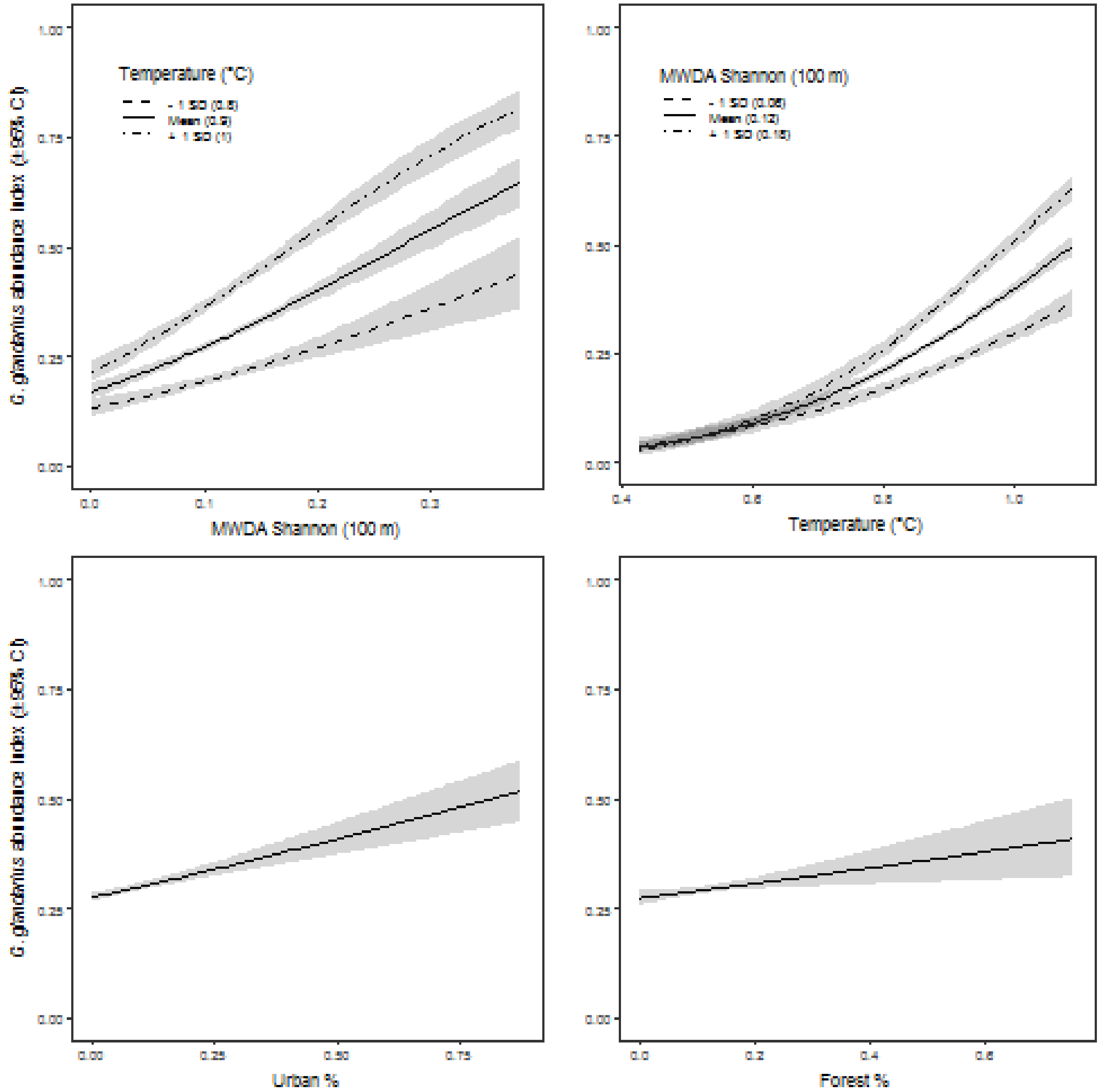

Figure AII.5 Main effect estimates for the full model.

We can also calculate the Johnson-Neyman interval to understand the range of moderator values for which the interaction term is significant. We then convert back to the measurement scale

```r
sim_slopes(jay_mod_best,
           pred = "shannon",
           modx = "bio1",
           johnson_neyman = TRUE)$jn
```

```
## [[1]]
## JOHNSON-NEYMAN INTERVAL
##
## When bio1 is OUTSIDE the interval [-6.84, -2.41], the slope of shannon
## is p < .05.
##
## Note: The range of observed values of bio1 is [-4.60, 1.91]
```

```r
# significant for -2.41 to 1.91 - HC'd so update if model changes
shannon_slope <- paste(10*round(c(-2.41, 1.91) *
                                  filter(means, covariate == "bio1") %>% pull(sd) +
                                  filter(means, covariate == "bio1") %>% pull(mean), 2),
                              collapse = ", ")

sim_slopes(jay_mod_best,
           pred = "bio1",
           modx = "shannon",
           johnson_neyman = TRUE)$jn
```

```
## [[1]]
## JOHNSON-NEYMAN INTERVAL
##
## When shannon is OUTSIDE the interval [-8.29, -3.28], the slope of bio1
## is p < .05.
##
## Note: The range of observed values of shannon is [-1.89, 4.18]
```

```r
# significant for full range of data - HC'd so update if model changes
temp_slope <- paste(round(c(-1.89, 4.18) *
                            filter(means, covariate == "shannon") %>% pull(sd) +
                            filter(means, covariate == "shannon") %>% pull(mean), 2),
                    collapse = ", ")
```

On the measurement scale, MWDA Shannon (1km) slope is significant in Temperature range 6.5, 10.9 (all but the lowest temperatures) and Temperature slope is significant in MWDA Shannon (1km) range 0, 0.38 (the full range of the data)

Collinearity diagnostics of the final model

We do not expect collinearity in the final model due to low correlations between variables, however we have included the variance inflation factor and condition index/variance decomposition tests to clarify.

Variance inflation factor

```r
vif(jay_mod_best) %>%
  kable(digits = 2, col.names = c("VIF"))
```

	VIF
shannon	2.33

	VIF
habitat	2.54
urban	1.11
bio1	1.30
shannon:bio1	1.06

Table AII.3 Variance inflation factors for each term in the final model.

Condition index and variance decomposition

```
mod_diag <- colldiag(jay_mod_best) %>%
  lapply(as.data.frame)
mod_diag <- do.call("cbind", mod_diag)
names(mod_diag) <- c("CI", "Intercept", "MWDA Shannon", "Forest %", "Urban %", "Temp")
kable(mod_diag, digits = 3)
```

CI	Intercept	MWDA Shannon	Forest %	Urban %	Temp
1.000	0	0.076	0.086	0.038	0.070
1.310	0	0.081	0.021	0.376	0.178
1.396	1	0.000	0.000	0.000	0.000
1.698	0	0.035	0.001	0.586	0.577
2.866	0	0.808	0.893	0.000	0.174

Table AII.4 Condition index and variance decomposition.

Session Info

```
session <- devtools::session_info()
session[[1]]
```

```
##  setting  value
##  version  R version 3.5.1 (2018-07-02)
##  os       Windows 10 x64
##  system   x86_64, mingw32
##  ui       RTerm
##  language (EN)
##  collate  English_United Kingdom.1252
##  ctype    English_United Kingdom.1252
##  tz       Europe/London
##  date     2019-02-15
```

```
session[[2]] %>% select(package, loadedversion, source) %>% kable
```

	package	loadedversion	source
abind	abind	1.4-5	CRAN (R 3.5.0)
assertthat	assertthat	0.2.0	CRAN (R 3.5.0)
backports	backports	1.1.2	CRAN (R 3.5.0)
base64enc	base64enc	0.1-3	CRAN (R 3.5.0)
bindr	bindr	0.1.1	CRAN (R 3.5.0)
bindrcpp	bindrcpp	0.2.2	CRAN (R 3.5.0)

	package	loadedversion	source
broom	broom	0.5.0	CRAN (R 3.5.1)
callr	callr	3.0.0	CRAN (R 3.5.1)
car	car	3.0-2	CRAN (R 3.5.1)
carData	carData	3.0-2	CRAN (R 3.5.1)
cellranger	cellranger	1.1.0	CRAN (R 3.5.0)
class	class	7.3-14	CRAN (R 3.5.1)
classInt	classInt	0.2-3	CRAN (R 3.5.0)
cli	cli	1.0.1	CRAN (R 3.5.1)
codetools	codetools	0.2-15	CRAN (R 3.5.1)
colorspace	colorspace	1.4-0	CRAN (R 3.5.2)
corrr	corrr	0.3.0	CRAN (R 3.5.1)
cowplot	cowplot	0.9.3	CRAN (R 3.5.1)
crayon	crayon	1.3.4	CRAN (R 3.5.0)
curl	curl	3.2	CRAN (R 3.5.0)
data.table	data.table	1.11.8	CRAN (R 3.5.1)
DBI	DBI	1.0.0	CRAN (R 3.5.0)
debugme	debugme	1.1.0	CRAN (R 3.5.0)
desc	desc	1.2.0	CRAN (R 3.5.0)
devtools	devtools	2.0.0	CRAN (R 3.5.1)
DHARMa	DHARMa	0.2.0	CRAN (R 3.5.1)
digest	digest	0.6.18	CRAN (R 3.5.1)
dplyr	dplyr	0.7.7	CRAN (R 3.5.1)
e1071	e1071	1.7-0	CRAN (R 3.5.1)
evaluate	evaluate	0.12	CRAN (R 3.5.1)
forcats	forcats	0.3.0	CRAN (R 3.5.0)
foreach	foreach	1.4.4	CRAN (R 3.5.0)
foreign	foreign	0.8-71	CRAN (R 3.5.1)
fs	fs	1.2.6	CRAN (R 3.5.2)
ggplot2	ggplot2	3.1.0	CRAN (R 3.5.1)
glue	glue	1.3.0.9000	Github (tidyverse/glue@3f7012c)
grainchanger	grainchanger	0.0.0.9000	local
gtable	gtable	0.2.0	CRAN (R 3.5.0)
haven	haven	1.1.2	CRAN (R 3.5.1)
highr	highr	0.7	CRAN (R 3.5.1)
hms	hms	0.4.2	CRAN (R 3.5.0)
htmltools	htmltools	0.3.6	CRAN (R 3.5.0)
httr	httr	1.3.1	CRAN (R 3.5.0)
iterators	iterators	1.0.10	CRAN (R 3.5.1)
jsonlite	jsonlite	1.6	CRAN (R 3.5.2)
jtools	jtools	1.1.1	CRAN (R 3.5.1)
knitr	knitr	1.21	CRAN (R 3.5.2)
labeling	labeling	0.3	CRAN (R 3.5.0)
lattice	lattice	0.20-35	CRAN (R 3.5.1)
lazyeval	lazyeval	0.2.1	CRAN (R 3.5.0)
lme4	lme4	1.1-18-1	CRAN (R 3.5.1)
lubridate	lubridate	1.7.4	CRAN (R 3.5.0)
magrittr	magrittr	1.5	CRAN (R 3.5.0)
MASS	MASS	7.3-50	CRAN (R 3.5.1)
Matrix	Matrix	1.2-14	CRAN (R 3.5.1)
memoise	memoise	1.1.0	CRAN (R 3.5.0)
minqa	minqa	1.2.4	CRAN (R 3.5.0)
modelr	modelr	0.1.2	CRAN (R 3.5.0)

	package	loadedversion	source
munsell	munsell	0.5.0	CRAN (R 3.5.1)
nlme	nlme	3.1-137	CRAN (R 3.5.1)
nloptr	nloptr	1.2.1	CRAN (R 3.5.1)
openxlsx	openxlsx	4.1.0	CRAN (R 3.5.1)
perturb	perturb	2.05	CRAN (R 3.5.0)
pillar	pillar	1.3.1	CRAN (R 3.5.2)
pkgbuild	pkgbuild	1.0.2	CRAN (R 3.5.1)
pkgconfig	pkgconfig	2.0.2	CRAN (R 3.5.1)
pkgload	pkgload	1.0.2	CRAN (R 3.5.2)
plyr	plyr	1.8.4	CRAN (R 3.5.0)
prettyunits	prettyunits	1.0.2	CRAN (R 3.5.0)
processx	processx	3.2.0	CRAN (R 3.5.1)
ps	ps	1.2.0	CRAN (R 3.5.1)
purrr	purrr	0.2.5	CRAN (R 3.5.1)
R6	R6	2.3.0	CRAN (R 3.5.1)
raster	raster	2.8-6	Github (rspatial/raster@589de57)
Rcpp	Rcpp	1.0.0	CRAN (R 3.5.2)
readr	readr	1.1.1	CRAN (R 3.5.0)
readxl	readxl	1.1.0	CRAN (R 3.5.0)
remotes	remotes	2.0.1	CRAN (R 3.5.1)
rio	rio	0.5.10	CRAN (R 3.5.0)
rlang	rlang	0.3.0.1	CRAN (R 3.5.1)
rmarkdown	rmarkdown	1.11	CRAN (R 3.5.2)
rprojroot	rprojroot	1.3-2	CRAN (R 3.5.0)
rstudioapi	rstudioapi	0.8	CRAN (R 3.5.1)
rvest	rvest	0.3.2	CRAN (R 3.5.0)
scales	scales	1.0.0	CRAN (R 3.5.1)
sessioninfo	sessioninfo	1.1.0	CRAN (R 3.5.1)
sf	sf	0.7-1	CRAN (R 3.5.1)
sp	sp	1.3-1	CRAN (R 3.5.1)
spData	spData	0.2.9.4	CRAN (R 3.5.1)
stringi	stringi	1.2.4	CRAN (R 3.5.1)
stringr	stringr	1.3.1	CRAN (R 3.5.0)
testthat	testthat	2.0.1	CRAN (R 3.5.1)
tibble	tibble	2.0.0	CRAN (R 3.5.1)
tidyr	tidyr	0.8.2	CRAN (R 3.5.1)
tidyselect	tidyselect	0.2.5	CRAN (R 3.5.1)
tidyverse	tidyverse	1.2.1	CRAN (R 3.5.0)
units	units	0.6-1	CRAN (R 3.5.1)
usethis	usethis	1.4.0	CRAN (R 3.5.2)
viridisLite	viridisLite	0.3.0	CRAN (R 3.5.0)
withr	withr	2.1.2	CRAN (R 3.5.1)
xfun	xfun	0.4	CRAN (R 3.5.1)
xml2	xml2	1.2.0	CRAN (R 3.5.0)
yaml	yaml	2.2.0	CRAN (R 3.5.1)
zip	zip	1.0.0	CRAN (R 3.5.1)
